# Unsuccessful Direct Acting Antiviral Hepatitis C Treatment Among People With HIV: Findings From an International Cohort

**DOI:** 10.1111/liv.16203

**Published:** 2024-12-10

**Authors:** Brendan L. Harney, Rachel Sacks‐Davis, Daniela K. van Santen, Ashleigh C. Stewart, Gail V. Matthews, Joanne M. Carson, Marina B. Klein, Karine Lacombe, Linda Wittkop, Dominque Salmon, Olivier Leleux, Laurence Merchadou, Marc van der Valk, Colette Smit, Maria Prins, Anders Boyd, Juan Berenguer, Inmaculada Jarrin, Andri Rauch, Margaret E. Hellard, Joseph S. Doyle

**Affiliations:** ^1^ Disease Elimination Program Burnet Institute Melbourne Australia; ^2^ School of Public Health and Preventive Medicine Monash University Melbourne Australia; ^3^ Department of Infectious Diseases Alfred Health and Monash University Melbourne Australia; ^4^ School of Population and Global Health University of Melbourne Melbourne Australia; ^5^ Department of Infectious Diseases, Research and Prevention Public Health Service of Amsterdam Amsterdam The Netherlands; ^6^ The Kirby Institute UNSW Sydney Australia; ^7^ St Vincent's Hospital Sydney Australia; ^8^ Division of Infectious Diseases and Chronic Viral Illness Service, Department of Medicine McGill University Health Centre Montreal Canada; ^9^ Sorbonne Université, INSERM, Institut Pierre Louis d'Épidémiologie et de Santé Publique Paris France; ^10^ Service de Maladies Infectieuses Hôpital Saint‐Antoine APHP Paris France; ^11^ CHU Bordeaux, Service d'information médicale Bordeaux France; ^12^ University of Bordeaux, INSERM, Bordeaux Population Health Research Centre U1219, CIC‐EC Bordeaux France; ^13^ INRIA SISTM Team Talence France; ^14^ Université Paris Descartes, Service Maladies Infectieuses et Tropicales, AP‐HP, Hôpital Cochin Paris France; ^15^ Department of Infectious Diseases Amsterdam University Medical Centers, University of Amsterdam Amsterdam Netherlands; ^16^ Amsterdam Infection & Immunity Institute, Amsterdam University Medical Centers, University of Amsterdam Amsterdam The Netherlands; ^17^ Stichting HIV Monitoring Amsterdam The Netherlands; ^18^ Centro de Investigación Biomédica en Red de Enfermedades Infecciosas (CIBERINFEC) Madrid Spain; ^19^ Infectious Diseases, Hospital General Universitario Gregorio Marañón (IsSGM) Madrid Spain; ^20^ Instituto de Salud Carlos III Madrid Spain; ^21^ Department of Infectious Diseases Inselspital, Bern University Hospital, University of Bern Bern Switzerland; ^22^ Doherty Institute and School of Population and Global Health University of Melbourne Melbourne Australia

**Keywords:** clinical research, direct acting antiviral, hepatitis C, HIV, treatment

## Abstract

**Background:**

Historically, hepatitis C virus (HCV) was difficult to treat among people with HIV. However, treatment with direct‐acting antivirals (DAAs) results in 90%–95% of people being cured. There is a need to understand why a proportion of people are not cured. We aimed to examine characteristics that may indicate an increased probability of unsuccessful DAA HCV treatment.

**Methods:**

Data were from the International Collaboration on Hepatitis C Elimination in HIV Cohorts. People who commenced DAA HCV treatment between 2014 and 2019 were included. Unsuccessful treatment was defined as a positive HCV RNA test at a person's first RNA test at least 4 weeks (SVR4+) following the end of treatment. Multivariable mixed‐effects logistic regression was used to examine characteristics associated with unsuccessful treatment.

**Results:**

Of 4468 people who commenced DAA treatment, 4098 (91.7%) had an SVR test 4+ weeks following the end of treatment, 207 (5%) of whom were unsuccessfully treated. Compared to a CD4+ cell count > 500 cells/mm^3^, cell counts < 200 (aOR 1.81, 95%CI 1.00–3.29) and between 200 and 349 (aOR 1.95, 95%CI 1.30–2.93) were associated with increased odds of unsuccessful treatment. Among 1921 people with data on injection drug use in the 12 months prior to treatment, there was some evidence that recent injection drug use was associated with increased odds of unsuccessful treatment; however, this was not statistically significant (aOR 1.67, 95%CI 0.99–2.82).

**Conclusions:**

The overwhelming majority of people were successfully treated for HCV. Overall, 5% of those with an SVR4+ test were unsuccessfully treated; this was more likely among people with evidence of immunodeficiency and those who reported recently injecting drugs.

**Abbreviations**
aOR: adjusted odds ratio
DAA: direct‐acting antiviral
HCV: hepatitis C virus
InCHEHC: International Collaboration on Hepatitis C Elimination in HIV Cohorts
RNA: ribonucleic acid
SVR: sustained virological response

**Summary**

- Historically, hepatitis C was difficult to treat among people with HIV; however, new treatments result in very high cure rates.
- Drawing on a large international cohort of people with HIV and hepatitis C, we found that 95% were successfully treated, while 5% were unsuccessfully treated.
- Unsuccessful treatment was more common among people with lower CD4+ cell counts and those who reported recently injecting drugs.

## 1 Background

Before the availability of direct‐acting antivirals (DAAs), treatment for hepatitis C virus (HCV) with interferon‐based regimes was sub‐optimal with only 50%–60% of people being cured and a range of side effects occurring [1]. Among people with HIV, successful HCV treatment was even less common, particularly among people with genotype 1, with 26%–40% being cured [2]. However, with interferon‐free DAA treatment, cure rates of approximately 95% are common in both clinical trials and real‐world settings [3, 4, 5, 6, 7]. While 95% of people with HIV/HCV being successfully treated is encouraging, there is a need to understand why a small proportion of these people are unsuccessfully treated. This is important at an individual level due to the potential development and/or progression of liver disease and for public health due to potential transmission, which may have implications for global targets of reductions in HCV‐related mortality and incidence, respectively [8].

A narrative review of HCV treatment among people with HIV reported cirrhosis to be a risk factor for unsuccessful treatment in some studies, while others did not [9]. Most of the studies were from Europe and conducted in the context of treatment prioritisation for people with advanced liver disease [10]. These studies were also often from hospital‐based specialist settings, with a liver cirrhosis prevalence that often approached, or exceeded, 50%. Cirrhosis was also associated with unsuccessful treatment in Canada, where 12% of people with cirrhosis were unsuccessfully treated [11]; similar results were reported among US military veterans, with 14% of people with cirrhosis unsuccessfully treated [4].

Many of these early studies did not include people with HIV/HCV actively injecting drugs and/or did not have these data available. In a Canadian study of 295 people who initiated DAA treatment up to July 2017, 8% did not achieve a sustained virological response (SVR) [11]. Among those who were defined as injecting drugs at a high frequency, unsuccessful treatment was slightly more common, with 11% not achieving a cure. A study from one US clinic and four European hospitals reported that among 784 people with HIV who commenced treatment up to the end of 2017, 7% were unsuccessfully treated [12]. As in the Canadian study, successful treatment was high across all groups (> 85%); however, ongoing substance use was associated with unsuccessful treatment. Conversely, a multi‐centre study of 642 people in the United States reported that 96.5% of people were successfully cured; there was no difference among people defined as using illicit drugs, with 96.4% cured [13].

Although these studies provide insight into unsuccessful HCV treatment, most had small sample sizes, which makes it challenging to understand factors associated with the critically important outcome of unsuccessful treatment. To understand the characteristics of people with HIV/HCV who are unsuccessfully treated, we examined unsuccessful direct‐acting antiviral treatment and associated behavioural, clinical and socio‐demographic predictors among people with HIV from an international cohort collaboration with data from Australia, Canada, France, The Netherlands, Spain and Switzerland.

## 2 Methods

### 2.1 Data Source

Data were obtained from the International Collaboration on Hepatitis C Elimination in HIV Cohorts (InCHEHC) [14]. InCHEHC has pooled data from cohorts of people with HIV using a protocol based on the HIV Cohort Data Exchange Protocol (HICDEP) [15]. Data were available from Australia, Canada, France, The Netherlands, Spain and Switzerland up to the end of 2019. Ethical approval was obtained by each cohort independently. Ethical approval for InCHEHC was obtained from the Alfred Hospital Ethics Committee (Ethics approval 662/18).

### 2.2 Inclusion Criteria

People with HIV/HCV who commenced interferon‐free DAA treatment between January 2014 and December 2019 were eligible for inclusion. We excluded people who did not have adequate follow‐up time to ascertain cure outcomes based on pre‐defined cohort database locks or the last date of data collection for each cohort. For example, if a person started treatment on 15th June 2019 and the last date of data collection for that cohort was 31st July 2019, they would have been excluded as this is only approximately 6 weeks. We only included a person's first interferon‐free DAA treatment and excluded any DAA treatment if there was a record of receiving interferon in the following 30 days. DAA treatments given in conjunction with interferon were also excluded (e.g., Boceprevir, Telaprevir), even if there was no record of interferon treatment.

### 2.3 Outcome Definitions

#### 2.3.1 Unsuccessful Treatment

**FIGURE 1:**
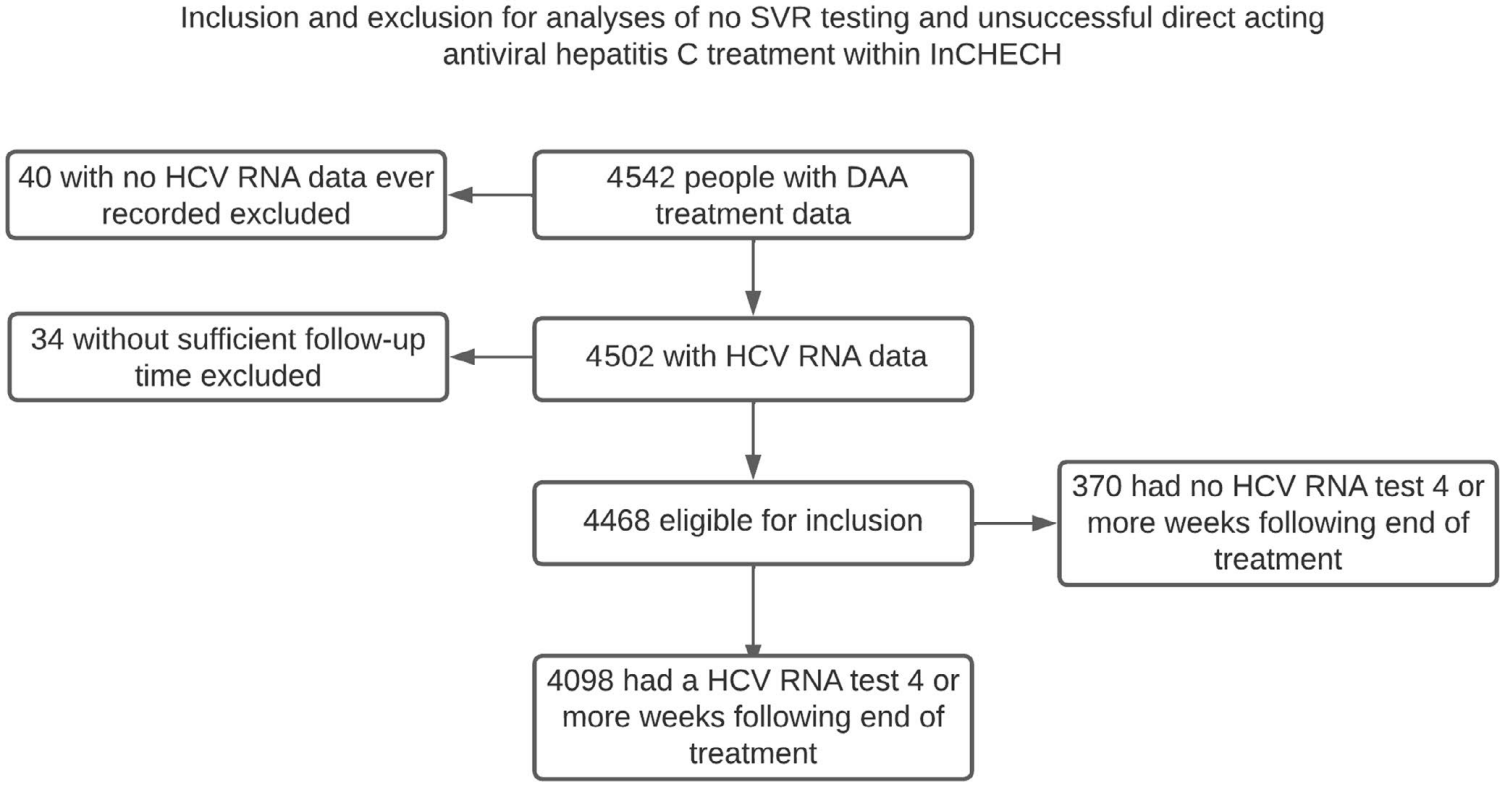
Flowchart of people included in analyses.

The primary outcome of unsuccessful treatment was defined as a detectable HCV viral load at a person's first SVR test 4 or more weeks (SVR4+) after the end of treatment (Supporting Information Methods: Figure 1). In a sensitivity analysis, we used SVR12+. SVR4 was used in the primary analysis as opposed to SVR12 because in real‐world settings, some people have an HCV RNA test at an earlier time point and no test thereafter. In addition, evidence shows very high concordance between SVR4 and SVR12 [16, 17, 18]. We defined a detectable viral load as a positive qualitative HCV RNA result and/or a quantitative result of HCV RNA viral load > 20 IU/mL. As unsuccessful treatment may be due to relapse or non‐response, we also examined the proportion attributable to each (Supporting Information Methods: Figure 1).

#### 2.3.2 No SVR4+ Test

The secondary outcome examined was no SVR test 4+ weeks after the end of treatment. Due to the variation in data collected across cohorts regarding loss to follow‐up, we included people who had no SVR4+ test, regardless of whether they were officially recorded as lost to follow‐up or not.

#### 2.3.3 Covariables at DAA Initiation

Covariables considered for inclusion in our analyses were age, key population group (gay and bisexual males, males with a history of injecting drug use, females with a history of injecting drug use, males with heterosexual or other exposure and females with heterosexual or other exposure), years since HIV diagnosis at DAA initiation, having an undetectable HIV viral load (HIV viral load < 200 copies/mL), CD4+ cell count, HCV genotype, possible cirrhosis, previous interferon treatment and known injection drug use within the last 12 months. We included these clinical and biological measures at the date closest to the start of treatment. If a measure was not available preceding treatment, we took the measure following treatment start provided it was within 14 days. Age, years since HCV diagnosis and years since HIV diagnosis were included as continuous variables in increments of 10 years. Further details on covariables are available in Supporting Information Methods.

#### 2.3.4 Missing Data

We used a multivariable multiple imputation fully conditional specified model to account for any potential bias due to missing data [19]. For the unsuccessful treatment analyses, we conducted this without imputation of the outcome; however, in a sensitivity analysis, we also imputed the outcome as has been done previously [13]. All variables, as applicable, including the cohort, were included in the multiple imputation models. For each analysis, 20 imputed datasets were created, and Von Hippel's two‐stage calculation procedure and related Stata command [20] were used to examine this, with more imputations added as required. Multiple imputation diagnostics were used to compare the distribution of observed to imputed variables [21]. Complete case analyses were also undertaken to compare and identify any variation in results from the multiple imputation analyses.

#### 2.3.5 Statistical Analyses

Characteristics potentially associated with unsuccessful treatment and no SVR test 4 or more weeks after the end of treatment were examined using two separate mixed‐effects logistic regression models, where the cohort was included as a random intercept. All covariables were included in multivariable analyses a priori. We included the key population groups as described in the primary analysis, as there may potentially be differences among these groups beyond sex, as has been shown in research on HCV treatment uptake from the same cohort [22]. In an additional analysis, we replaced this with a variable for sex at birth and GBM compared to all other groups.

HCV DAA treatment type was not included in the primary analysis, as this may have been in part influenced by other clinical factors already included, e.g., HCV genotype and cirrhosis. We did however examine this in an additional multivariable analysis to understand how this may have confounded our results from the primary analysis. Due to the different availability of data to examine possible cirrhosis, we also conducted an additional analysis whereby possible cirrhosis was defined solely based on transient elastography data. For the unsuccessful treatment outcome, we also examined whether there was any difference in results when SVR12+ was used instead of SVR4+.

An additional analysis was conducted to examine recent injection drug use and unsuccessful treatment. To avoid potential issues with multicollinearity in this analysis, we replaced the key population group variable with a variable for sex at birth and a variable for gay and bisexual men, compared to all other groups.

We also conducted analyses whereby the variables for age, CD4+ cell count, years since HIV diagnosis and years since first HCV‐positive test were modelled as restricted cubic splines due to potential issues with categorisation of continuous variables [23]. Four knots were placed corresponding to the 20th, 40th, 60th and 80th percentile. The Stata command *xblc* and related publication [24] were used to guide these analyses and interpretation. For CD4+ cell count, 500 was used as the reference value. The mean was used for the other variables: 50 years for age, 15 years for years living with HIV and seven for years since HCV diagnosis.

All analyses were undertaken using Stata/SE 18.0 (College Station, Texas, United States of America).

## 3 Results

Overall, 4542 people had DAA treatment data recorded, of whom 4502 (99.1%) had HCV RNA and/or SVR‐related data recorded at least once. Among these 4502 people, 4468 (99.2%) had sufficient follow‐up time for outcome ascertainment (Figure 1). The majority of these 4468 people were male (81.3%), and the mean age was 50 (range 21–85, SD 8.9). Almost half were defined as gay or bisexual males (44.4%), and approximately one‐quarter were males with a history of injection drug use (25.6%) (Table 1). Slightly more than half had HCV genotype 1 (56.7%), approximately 18% were defined as having possible cirrhosis, and one‐quarter (25.6%) had a record of previous interferon‐based treatment. The majority had a CD4+ cell count greater than 500 cells/mm^3^ (61.0%) and were virologically suppressed regarding HIV (91.0%). Sofosbuvir/Ledipasvir was the most commonly prescribed treatment (43.3%). The majority of these people, 91.7% (*n* = 4098), had an SVR4+ test (Supporting Information Results: Table 1). Information related to missing data is available in Supporting Information Results: Table 2.

**TABLE 1:** Demographic, behavioural and clinical characteristics overall and by treatment outcomes among 4098 people with DAA treatment data and an SVR4+ test.

	Overall (*N* = 4468)	Treatment outcome (*N* = 4098)	
		Unsuccessful treatment (*n* = 207)	Successful treatment (*n* = 3891)
Age, mean (SD, range)^a^	50 (8.9, 21–85)	50 (9.3, 24–75)	50 (8.8, 21–85)
Country			
Australia	462 (10.3)	18 (4.1)	421 (95.9)
Canada	703 (15.7)	52 (7.9)	603 (92.1)
France	880 (19.7)	44 (5.4)	771 (94.6)
The Netherlands	1026 (23.0)	37 (3.8)	942 (96.2)
Spain	585 (13.1)	21 (4.5)	448 (95.5)
Switzerland	812 (18.2)	35 (4.7)	706 (95.3)
Population group^b^			
GBM	1986 (44.4)	79 (4.3)	1775 (95.7)
Male Hx of IDU	1145 (25.6)	68 (6.6)	965 (93.4)
Female Hx of IDU	491 (11.0)	13 (2.9)	435 (97.1)
Male hetero/other	503 (11.3)	30 (6.7)	421 (93.3)
Female hetero/other	333 (7.5)	17 (5.6)	285 (94.4)
Unknown/missing	10 (0.2)		10 (100.0)
Recent injection drug use^c^			
No	1556 (34.8)	68 (4.7)	1370 (95.3)
Yes	365 (8.2)	26 (7.6)	316 (92.4)
Unknown/missing	2547 (57.0)	113 (4.9)	2205 (95.1)
Genotype^d^			
GT1	2533 (56.7)	104 (4.4)	2237 (95.6)
GT2	126 (2.8)	9 (7.8)	106 (92.2)
GT3	657 (14.7)	34 (5.7)	563 (94.3)
GT4	673 (15.1)	37 (6.0)	581 (94.0)
GT5	1 (0.0)	0	1 (100.0)
GT6	6 (0.1)	0	6 (100.0)
Unknown/missing	472 (10.6)	23 (5.5)	397 (94.5)
CD4+ cell count^d^			
500+	2726 (61.0)	101 (4.0)	2407 (96.0)
499–350	757 (16.9)	35 (5.0)	666 (95.0)
200–349	518 (11.6)	38 (8.2)	428 (91.8)
< 200	247 (5.5)	17 (7.8)	201 (92.2)
Unknown/missing	220 (4.9)	16 (7.8)	189 (92.2)
Transient elastography score^c^, ^d^			
< 12.5	2544 (56.9)	106 (4.5)	2247 (95.5)
12.5–21	302 (6.8)	18 (6.3)	268 (93.7)
≥ 21	301 (6.7)	20 (7.4)	250 (92.6)
Unknown/missing	1321 (29.6)	63 (5.3)	1126 (94.7)
FIB‐4 score^c^, ^d^			
≤ 3.25	2864 (64.1)	113 (4.3)	2505 (95.7)
> 3.25	546 (12.2)	31 (6.3)	462 (93.7)
Unknown/missing	1058 (23.7)	63 (6.4)	924 (93.6)
Possible cirrhosis^e^			
No	3467 (77.6)	143 (4.5)	3043 (95.5)
Yes	780 (17.5)	50 (7.0)	664 (93.0)
Unknown/missing	221 (4.9)	14 (7.1)	184 (92.9)
Years since HIV diagnosis			
< 10	1346 (30.1)	62 (5.1)	1159 (94.9)
10–19	1429 (32.0)	68 (5.2)	1231 (94.8)
20–29	1293 (28.9)	58 (4.8)	1158 (95.2)
30+	357 (8.0)	13 (4.0)	308 (96.0)
Unknown/missing	43 (1.0)	6 (14.6)	35 (85.4)
Years since first HCV positive test			
< 10	2663 (59.6)	122 (5.0)	2318 (95.0)
10–19	1231 (27.6)	60 (5.3)	1069 (94.7)
20+	518 (11.6)	25 (5.2)	453 (94.8)
Unknown/missing	56 (1.3)		51 (100.0)
HIV RNA viral load^d^			
< 200 copies/mL	4066 (91.0)	181 (4.8)	3559 (95.2)
≥ 200 copies/mL	203 (4.5)	11 (6.5)	159 (93.5)
Unknown/missing	199 (4.5)	15 (8.0)	173 (92.0)
Previous HCV treatment^f^			
No	3316 (74.2)	145 (4.8)	2861 (95.2)
Yes	1152 (25.8)	62 (5.7)	1030 (94.3)
DAA prescription			
Sofosbuvir	179 (4.0)	24 (14.3)	144 (85.7)
Sofosbuvir/Ledipasvir	1934 (43.3)	73 (4.0)	1738 (96.0)
Sofosbuvir/Velpatasvir	577 (12.9)	29 (6.2)	437 (93.8)
Elbasvir/Grasoprevir	330 (7.4)	21 (6.9)	282 (93.1)
Sofosbuvir/Daclatasvir	685 (15.3)	29 (4.5)	618 (95.5)
Paritaprevir/Ombitasvir/Ritonavir ± Dasabuvir	453 (10.1)	17 (4.0)	413 (96.0)
Glecaprevir/Pibrentasvir	156 (3.5)	6 (4.7)	121 (95.3)
Sofosbuvir/Velpatasvir/Voxilaprevir	6 (0.1)		5 (100.0)
Sofosbuvir/Simeprevir	148 (3.3)	8 (5.7)	133 (94.3)

*Note: n* (%) unless otherwise stated; overall % reflects column, treatment % reflects rows. Data comparing people with and without an SVR4+ test are available in Supporting Information Results: Table 1.

^a^ At the time of treatment start.

^b^ Based on HIV and/or HCV exposure data and sexuality data, gay and bisexual males who also have a history of IDU are classified as gay and bisexual males. Other includes exposures such as perinatal, transfusion and accidents where a history of IDU or GBM is not known from the data available.

^c^ These data were never collected by some cohorts.

^d^ At the prior date closest to treatment or within 14 days of treatment start if no prior data were recorded.

^e^ Defined by a composite of transient elastography (12.5 kPa) and FIB‐4 score (3.25). Where both were available, the one closest to treatment start was used. No liver biopsy data were available.

^f^ Based on the record of previous interferon treatment, including combined DAA/interferon treatment, i.e., boceprevir, telaprevir. People without these data recorded are classified as ‘no’ rather than ‘unknown’, as the absence of previous treatment is not recorded for any cohort.

### 3.1 Unsuccessful Treatment

Among the 4098 people with an SVR4+ test recorded, 207 (5.0%) were defined as unsuccessfully treated. Of the 207, 129 (62.3%) were defined as relapse, and 78 (37.7%) were defined as non‐response. There was modest variation in unsuccessful treatment across cohorts, ranging from 2.3% to 7.9%. In multivariable analysis, compared to a CD4+ cell count > 500 cells/mm^3^, a cell count < 200 (aOR 1.81, 95%CI 1.00–3.29) and between 200 and 349 (aOR 1.95, 95%CI 1.30–2.93) were each associated with increased odds of unsuccessful treatment (Table 2). Genotype four was also associated with increased odds of unsuccessful treatment compared to genotype one (aOR 1.56, 95%CI 1.04–2.32).

**TABLE 2:** Unsuccessful treatment among people with an SVR4+ test: *n*(%), odds ratio (OR), adjusted OR (aOR) and 95% confidence interval (95%CI).

	OR (95%CI)	aOR (95%CI)
Age (10 years)	1.03 (0.88–1.20)	1.00 (0.85–1.19)
Population group		
GBM	1.00	1.00
Male Hx of IDU	1.44 (1.01–2.06)	1.37 (0.92–2.03)
Female Hx of IDU	0.60 (0.33–1.11)	0.59 (0.31–1.12)
Male hetero/other	1.52 (0.98–2.36)	1.39 (0.88–2.19)
Female hetero/other	1.25 (0.72–2.17)	1.11 (0.63–1.97)
Previous HCV treatment		
No	1.00	1.00
Yes	1.21 (0.88–1.67)	1.22 (0.87–1.71)
CD4+ cell count		
500+	1.00	1.00
499–350	1.26 (0.84–1.89)	1.24 (0.83–1.87)
200–349	2.04 (1.37–3.03)	1.95 (1.30–2.93)
< 200	1.86 (1.04–3.33)	1.81 (1.00–3.29)
Possible cirrhosis		
No	1.00	1.00
Yes	1.47 (1.05–2.06)	1.27 (0.89–1.83)
Genotype		
GT1	1.00	1.00
GT2	1.78 (0.89–3.56)	1.79 (0.88–3.62)
GT3	1.25 (0.83–1.86)	1.16 (0.77–1.74)
GT4	1.44 (0.96–2.14)	1.56 (1.04–2.32)
Years since HIV diagnosis		
< 10	1.00	1.00
10–19	0.98 (0.69–1.40)	0.89 (0.60–1.33)
20–29	0.85 (0.57–1.25)	0.69 (0.42–1.12)
30+	0.74 (0.40–1.39)	0.64 (0.32–1.29)
Years since the first HCV‐positive test		
< 10	1.00	1.00
10–19	1.10 (0.79–1.54)	1.08 (0.72–1.64)
20+	1.08 (0.67–1.75)	1.16 (0.64–2.11)
HIV RNA viral load		
< 200 copies/mL	1.00	1.00
≥ 200 copies/mL	1.27 (0.66–2.43)	1.09 (0.56–2.11)

In the group of 1921 people with data on injection drug use within the 12 months prior to treatment, 1780 (92.7%) had an SVR4+ test, of whom 94 (5.3%) were unsuccessfully treated. In the multivariable analysis, there was weak evidence of an association between recent injection drug use increased odds of unsuccessful treatment (aOR 1.67, 95%CI 0.99–2.82); however, the 95% confidence interval for the aOR included one, suggesting that the data were also consistent with no association. A CD4+ cell count between 200 and 349 was also associated with a 2‐fold increase in the odds of unsuccessful treatment (aOR 2.04, 95%CI 1.15–3.64), and compared to male sex, female sex was protective against unsuccessful treatment (aOR 0.45, 95%CI 0.23–0.88) (Table 3).

**TABLE 3:** Unsuccessful treatment among people with data on recent injecting drug use and a SVR4+ test: *n* (%), OR, aOR and 95% confidence Interval (95%CI).

	OR (95%CI)	aOR (95%CI)
Age (10 years)	1.13 (0.89–1.43)	1.13 (0.87–1.48)
Sex at birth		
Male	1.00	1.00
Female	0.44 (0.24–0.82)	0.45 (0.23–0.88)
Gay or bisexual male		
Yes	1.00	1.00
No	0.81 (0.52–1.26)	0.99 (0.59–1.66)
Recent injection drug use		
No	1.00	1.00
Yes	1.52 (0.92–2.52)	1.67 (0.99–2.82)
Previous HCV treatment		
No	1.00	1.00
Yes	1.25 (0.75–2.07)	1.25 (0.74–2.13)
CD4+ cell count		
500+	1.00	1.00
499–350	1.16 (0.67–2.03)	1.13 (0.64–1.99)
200–349	1.88 (1.08–3.30)	2.04 (1.15–3.64)
< 200	1.40 (0.63–3.09)	1.57 (0.69–3.55)
Possible cirrhosis		
No	1.00	1.00
Yes	1.15 (0.69–1.91)	1.03 (0.60–1.78)
Genotype		
GT1	1.00	1.00
GT2	0.89 (0.21–3.75)	0.92 (0.21–3.98)
GT3	1.11 (0.63–1.94)	1.11 (0.62–1.97)
GT4	1.39 (0.75–2.59)	1.70 (0.90–3.22)
Years since HIV diagnosis		
< 10	1.00	1.00
10–19	1.29 (0.74–2.25)	1.20 (0.65–2.21)
20–29	0.99 (0.54–1.80)	0.95 (0.47–1.90)
30+	1.01 (0.43–2.37)	1.08 (0.42–2.76)
Years since first HCV positive test		
< 10	1.00	1.00
10–19	1.09 (0.66–1.78)	1.07 (0.60–1.89)
20+	0.63 (0.27–1.47)	0.62 (0.24–1.65)
HIV RNA viral load		
< 200 copies/mL	1.00	1.00
≥ 200 copies/mL	1.02 (0.40–2.60)	0.83 (0.32–2.17)

### 3.2 No SVR 4+ Test

Of the 4468 people eligible for inclusion, 370 (8.3%) did not have an SVR4+ test. Across the cohorts, this ranged from 3.2% to 19.8%. In multivariable analyses, an HIV viral load ≥ 200 copies/mL (aOR 1.98, 95%CI 1.31–2.99) was associated with increased odds of no SVR test (Table 4). Conversely, people with previous HCV treatment recorded were more likely to have an SVR test (aOR 0.66, 95%CI 0.48–0.90).

**TABLE 4:** No SVR test 4 or more weeks after the end of treatment among people who started treatment, *n* (%), OR, aOR and 95% confidence interval (95%CI).

	OR (95%CI)	aOR (95%CI)
Age (10 years)	0.91 (0.81–1.03)	0.89 (0.78–1.02)
Population group		
GBM	1.00	1.00
Male Hx of IDU	1.27 (0.96–1.68)	1.28 (0.93–1.75)
Female Hx of IDU	1.24 (0.85–1.80)	1.19 (0.79–1.78)
Male hetero/other	1.44 (1.01–2.03)	1.42 (0.99–2.04)
Female hetero/other	1.23 (0.80–1.87)	1.14 (0.73–1.76)
Previous HCV treatment		
No	1.00	1.00
Yes	0.63 (0.47–0.86)	0.66 (0.48–0.90)
CD4+ cell count		
500+	1.00	1.00
499–350	0.91 (0.67–1.24)	0.86 (0.63–1.18)
200–349	1.32 (0.95–1.84)	1.19 (0.85–1.68)
< 200	1.58 (1.03–2.43)	1.34 (0.86–2.11)
Possible cirrhosis		
No	1.00	1.00
Yes	1.14 (0.85–1.52)	1.13 (0.83–1.53)
Genotype		
GT1	1.00	1.00
GT2	1.34 (0.72–2.53)	1.39 (0.73–2.63)
GT3	1.23 (0.91–1.67)	1.17 (0.86–1.61)
GT4	0.98 (0.70–1.37)	1.01 (0.71–1.42)
Years since HIV diagnosis		
< 10	1.00	1.00
10–19	1.13 (0.86–1.47)	1.21 (0.89–1.64)
20–29	0.76 (0.55–1.05)	0.80 (0.55–1.19)
30+	1.21 (0.80–1.82)	1.23 (0.76–2.00)
Years since the first HCV‐positive test		
< 10	1.00	1.00
10–19	1.00 (0.77–1.31)	1.01 (0.73–1.38)
20+	1.07 (0.72–1.58)	1.18 (0.74–1.88)
HIV RNA viral load		
< 200 copies/mL	1.00	1.00
≥ 200 copies/mL	2.10 (1.40–3.15)	1.98 (1.31–2.99)

### 3.3 Sensitivity Analyses

There was minimal difference in other results when sex at birth and GBM status were used in place of the population group variable (Supporting Information Results: Table 3). When HCV DAA treatment was added to the multivariable analysis, there was minimal difference with regards to CD4+ cell counts. However, as anticipated, this led to the association between genotype and unsuccessful treatment being weakened (Supporting Information Results: Table 4). Results were not sensitive to changes in the definition of possible cirrhosis based on transient elastography scores, which also included a category of > 21 kPa; removing the CD4+ cell count covariable made little difference (Supporting Information Results: Table 5). When SVR12+ was used instead of SVR4+, 217 people were defined as unsuccessfully treated and 533 did not have an SVR12+ test; there was minimal difference in the results from the multivariable analyses (Supporting Information Results: Table 6).

Using restricted cubic splines, our findings were consistent with the primary analysis. There was evidence that at CD4+ cell counts less than 500, the odds of unsuccessful treatment increased as CD4+ cell counts decreased (Supporting Information Results: Figure 1). There was no evidence that age, years living with HIV and years since the first HCV‐positive test were associated with unsuccessful treatment (Supporting Information Results: Figures 2–4).

Of the 207 people defined as unsuccessfully treated, 64 (30.9%) had genotype data available following their treatment start date. Among these 64 people, 11 had a different genotype and/or sub‐type following their treatment. When these people were excluded from analysis, there was some modest variation in findings; a CD4+ cell count 200–349 remained associated with unsuccessful treatment (aOR 1.96, 95%CI 1.30–2.96); however, the evidence of association with a CD4+ cell count < 200 was weakened (aOR 1.65, 95%CI 0.91–3.00). Likewise, the association with recent injection drug use was weakened (aOR 1.63, 95%CI 0.95–2.81).

### 3.4 Missing Data

These results did not appear to be substantially affected by missing data. Multiple imputation diagnostics indicated that there was little difference in the distributions of observed and imputed variables for both the unsuccessful treatment and no SVR4+ test analyses (Supporting Information Results: Table 7). Moreover, for the primary analysis of unsuccessful treatment, the results were consistent when the treatment outcome was imputed for those who had no SVR4+ test and in a complete case analysis (Supporting Information Results: Table 8). Likewise, results were similar in the secondary analysis focused on recent injection drug use (Supporting Information Results: Table 9). There was also minimal difference between the complete case and multiple imputation analysis for SVR4+ testing (Supporting Information Results: Table 10).

## 4 Discussion

In our international cohort collaboration of 4468 people with HIV who received DAA HCV treatment, 3891 (87.1%) were known to be successfully treated, 207 (4.6%) were unsuccessfully treated and 370 (8.3%) did not have an SVR4+ test to determine their treatment outcome. Among the 4098 people who did have an SVR4+ test, unsuccessful HCV treatment was uncommon with 5% of people having a positive HCV RNA result. As anticipated, 95% of people being successfully treated aligns with findings from clinical trials and other observational studies of people living with HIV [3, 4, 13]. While encouraging, understanding why approximately 5% of people are not successfully treated is a critical undertaking as people who are unsuccessfully treated may be at risk of developing or worsening liver disease. Unsuccessfully treated HCV may also inadvertently lead to new transmissions, including reinfections. Both of these may have implications for global targets to reduce HCV incidence and HCV‐related mortality [8].

We found that unsuccessful treatment was associated with low CD4+ cell counts. It is important to consider whether, in our context, people with HIV in high‐income countries with, for the most part, universal healthcare systems, low CD4+ cell counts represent sub‐optimal HIV treatment adherence, which in turn may have implications regarding HCV medication adherence. If adherence were the issue, we might expect to see a stronger relationship also between HIV viral load and unsuccessful treatment, which we did not. Lower CD4+ cell counts could also reflect a longer duration of HIV infection and/or start HIV treatment when CD4+ cell counts were already very low [25]. HIV/HCV co‐infection has also been associated with a lower level of immune restoration among people who start highly active antiretroviral therapy, with CD4+ cell counts sometimes remaining low despite HIV suppression [26, 27]. However, the association between unsuccessful treatment and lower CD4+ cell counts was independent of both HIV and HCV duration. It is also possible that our findings related to CD4+ cell counts are at least partially a result of liver disease rather than being related to HIV specifically. In studies focused on the roles of absolute CD4+ cell counts and CD4% in clinical decision‐making for HIV, there is evidence that liver fibrosis and/or cirrhosis may play a role in lower absolute CD4+ cell counts and discordance with CD4% [28, 29]. Lending credence to this are findings from a study among people without HIV, where cirrhosis was also associated with a lower CD4+ cell count [30]. Cirrhosis itself has been reported to be associated with an increased risk of unsuccessful treatment; however, this finding is not universal [9]. In our analyses, unsuccessful treatment was slightly more common among people with possible cirrhosis; however, we did not find this association in multivariable analyses. In their narrative review, Bischoff and Rockstroh [9] postulate that variations in unsuccessful treatment with regards to cirrhosis are likely due to differing prevalence and severity of cirrhosis among various cohorts and the treatments used. The prevalence of cirrhosis in our study was approximately 18% based on liver stiffness scores and/or FIB‐4 scores. It is possible that earlier studies, many with a high prevalence of cirrhosis due to treatment prioritisation and/or specialist settings, had many people with more advanced cirrhosis, which may reduce the probability of treatment success. This is supported by a Spanish study [31], which found that among people with cirrhosis, those with a liver stiffness score of 21 kPa, itself a marker of portal hypertension [32, 33], had 85% success compared with 93% among all people with cirrhosis. While unsuccessful treatment was more common among people who had a kPa score > 21 in our study, we did not find this association in multivariable analysis, including when the variable for CD4+ cell count was removed.

In a German study of 437 people with HIV and hepatitis C, no significant relationship was found between unsuccessful treatment and cirrhosis nor current absolute CD4+ cell counts [34]. Another German study found that baseline CD4+ cell count less than 350, CD4% < 20 and cirrhosis were associated with unsuccessful treatment in univariable analysis; however, only cirrhosis was statistically significant in multivariable analyses [35]. Despite CD4+ cell count not being statistically significant in multivariable analysis, the authors discuss the relationship between CD4+ cell counts and cirrhosis. They show that unsuccessful HCV treatment was more common among people with a CD4+ cell count less than 350, among both people with and without cirrhosis, and suggest that a low CD4+ cell could likely play a role in unsuccessful HCV treatment. A study from Spain, which included almost 2400 people [36], reported both cirrhosis and a CD4+ cell count < 200 to be associated with increased odds of unsuccessful treatment in multivariable analysis.

We also found that genotype four was associated with increased odds of unsuccessful treatment. As summarised in a review of genotype four treatment [37], successful DAA treatment among people with genotype four usually occurs in more than 90% of people, with the lower levels of successful treatment likely driven by liver disease and/or treatment with DAA regimes that in retrospect are somewhat less effective than current treatment regimes. It is conceivable that this is likely what is also driving our finding regarding genotype four.

Though not statistically significant, there was some evidence that recent injecting drug use was associated with an increase in the risk of unsuccessful treatment. However, it is important to note that 92% of people who self‐reported recent injection drug use were successfully treated. These results are consistent with findings from a US‐European collaboration [12], where ‘ongoing drug use’ and Canada [11], where ‘high‐frequency injection drug use’ were associated with unsuccessful treatment. Many of the people in the aforementioned Canadian study were included in our analysis; as noted by the authors of that study, the relationship between injecting drug use and unsuccessful treatment is potentially driven, at least to some degree by sub‐optimal adherence. Though not specific to people with HIV, this is also supported by other studies focused specifically on people who inject drugs [38]. While predicting future adherence is challenging, our results suggest that extra support during HCV treatment, tailored to individual needs, may be warranted among some people who inject drugs to help them achieve HCV cure.

While not having an SVR test is not the equivalent of unsuccessful treatment, it is still useful to consider differences between who is and is not having their treatment outcomes assessed, as it is possible that a higher proportion of people who do not take all their treatment, and hence are less likely to clear infection, also do not get an SVR test. In our multivariable analysis, previous interferon‐based HCV treatment was associated with having an SVR4+ test. Given the sub‐optimal treatment outcomes from interferon‐based treatment, as noted previously, it is possible that people who experienced interferon treatment were particularly interested in finding out if they had been cured with DAA treatment. It is also possible that people who were previously treated were more likely to have liver disease and/or other health issues and therefore were more closely followed up. We also found that no SVR test was associated with a detectable HIV viral load. While some people cannot achieve an undetectable HIV viral load despite being adherent to HIV treatment, a detectable HIV viral load may also be reflective of lower engagement in clinical care, providing less opportunity for SVR testing. Based on these findings, it would appear that there is minimal indication that our findings regarding unsuccessful treatment would be biased by the exclusion of people who did not have an SVR test. In addition, our analyses with the outcome imputed where people did not have an SVR4+ test were very similar to the analyses without this imputed.

There are some limitations to our analyses. First, as with many real‐world studies, data on adherence were not captured in a routine manner, and we were not able to assess this systematically across cohorts. However, it is also important to consider that while adherence would play a role in being successfully treated, imperfect adherence does not always equate to unsuccessful treatment [38, 39]. Relatedly, most cohorts did not have data on structural determinants of health, such as housing, which may play a role in adherence and engagement in care more generally, thus we are not able to examine how this may have influenced our findings. Second, differentiating between unsuccessful treatment due to relapse and reinfection post‐treatment, but before SVR is confirmed, is an ongoing challenge, and some unsuccessful treatment may have been reinfection. Previous studies have used a switch in genotype and/or subtype as a definition of reinfection [40, 41]. In our data, post‐treatment genotyping among people with evidence of unsuccessful treatment was uncommon, limiting our ability to examine this systematically. Even if these data were available, a lack of genotype switch does not rule out possible reinfection [42], as the same HCV genotype and subtype are common among sub‐groups of people potentially at risk of reinfection [43]. While this could be overcome with more in‐depth phylogenetic analyses [44], we did not have these data available. Third, with these data being pooled retrospectively, the level of detail on behaviours and recall periods across cohorts varied, including for recent injection drug use. Most cohorts and studies were predominantly designed to understand clinical outcomes rather than behavioural outcomes. As such, we were unable to examine the impact of most current behaviours on being unsuccessfully treated. This is a well‐recognised issue in other similar work among people with HIV [45]. Finally, all data were from high‐income countries with, generally speaking, universal health care systems; therefore, these data may not be generalisable to low‐ and middle‐income countries or those without universal health care systems.

## 5 Conclusions

In this large, international multi‐cohort collaboration, consistent with clinical trials and other observational studies, approximately 5% of people living with HIV who commenced DAA treatment had unsuccessful HCV treatment. While uncommon, unsuccessful treatment was associated with low absolute CD4+ cell counts and recent injection drug use. Extra support and monitoring through HCV treatment may be warranted among some people who report recent injection drug use and people with low CD4+ cell counts.

## Author Contributions

Conceptualisation and methodology: B.L.H., R.S.D., M.E.H. and J.S.D. Statistical analysis and writing of the original draft: B.L.H. Interpretation: B.L.H, R.S.‐D., M.E.H. and J.S.D. Data collection: J.M.C., M.B.K., K.L., L.W., D.S., O.L., L.M., M.V., C.S., M.P., A.B., J.B., I.J., A.R. and J.S.D. Data curation: D.K.S. and A.C.S. Funding acquisition: M.E.H. Reviewing and editing manuscript: all authors.

## Ethics Statement

Ethics approval for the coordinating centre was granted by the Alfred Hospital Human Research Ethics Committee. Ethics approval for each cohort has been granted by the following committees: ACCESS and Co‐EC: Alfred Hospital Human Research Ethics Committee. ANRS CO13 HEPAVIH: CPP Ile de France III. ANRS CO3 AQUITAINE: CPP Sud‐Ouest et Outre‐mer III. ATHENA cohort: At initiation, the cohort was approved by the institutional review board of all participating centres. People entering HIV care receive written material about participation in the ATHENA cohort and are being informed by their treating physician of the purpose of collection of data, after which they can consent verbally or elect to opt out. Data are pseudonymised before being provided to investigators and may be used for scientific purposes. A designated quality management coordinator safeguards compliance with the European General Data Protection Regulation. Canadian Coinfection Cohort: McGill University Health Centre Research Ethics Board. CEASE: St Vincent's Hospital Human Research Ethics Committee. CoRIS: Comité Ético de Investigación Clínica del Hospital General Universitario Gregorio Marañon. MOSAIC: Institutional Review Board of the Academic Medical Center and ethical committees/board of directors of each institute recruiting participants. SAIDCC: Registre général des traitements de l'APHP. SHCS: The SHCS was approved by the local ethical committees of the participating centres, and written informed consent was obtained from all participants (https://shcs.ch/206‐ethic‐committee‐approval‐and‐informed‐consent).

## Consent

Patient consent was undertaken in accordance with local regulatory guidelines as applicable for each cohort as part of the above‐noted ethics approvals.

## Conflicts of Interest

Juan Berenguer reports honoraria for advice or public speaking from AbbVie, Gilead, MSD, JANSSEN and ViiV Healthcare; and grants from AbbVie, Gilead, MSD and ViiV Healthcare. Marina Klein reports grants for investigator‐initiated studies from ViiV Healthcare, AbbVie and Gilead, and consulting fees from ViiV Healthcare, AbbVie and Gilead, all outside the submitted work. Andri Rauch reports support to his institution for advisory boards and/or travel grants from Abbvie, MSD, Gilead Sciences and Pfizer, and an investigator‐initiated trial grant from Gilead Sciences. All remuneration to Andri Rauch went to his home institution and not to Andri Rauch personally, and all remuneration was provided outside the submitted work. Karine Lacombe reports honoraria for advice or public speaking from Abbvie, Gilead, MSD, Janssen and ViiV Healthcare. Maria Prins reports unrestricted research grants and speaker/advisor fees from Gilead Sciences and MSD; all of which were paid to her institution and unrelated to the current work. Marc van der Valk reports unrestricted research grants and fees for participation in advisory boards from Gilead, MSD and ViiV (all paid to his institution). Joseph S Doyle reports funding to his institution for investigator‐initiated research from Gilead Sciences, Abbvie and BMS, and honoraria to his institution for educational events from AbbVie. Linda Wittkop reports grants/financial support for the work under consideration from the French Agency ANRS Emerging Infectious Diseases (ANRS—MIE) paid to her institution. Gail Matthews reports grants from Gilead, AbbVie and ViiV, all paid to her institution and financial support for participating in the advisory board from Gilead and ViiV. The CEASE study is supported by Gilead. Inmaculada Jarrin reports grants from MSD and ViiV Healthcare, all paid to her institution; honoraria for lectures/presentations from Gilead and ViiV Healthcare; support from Gilead for attending meetings/travel; and support from Gilead to participate in an advisory board. Margaret Hellard reports investigator‐initiated research grants from Gilead and Abbvie. Dominique Salmon reports support for attending meetings/travel from Gilead and AbbVie. All other authors have no relevant disclosures.

## Supporting information


Data S1.

## Data Availability

Data are available upon request from the InCHEHC steering committee. Initial requests should be directed to Rachel Sacks‐Davis (rachel.sacks-davis@burnet.edu.au).
