# Supplementary material for: Unsuccessful Direct Acting Antiviral Hepatitis C Treatment Among People With HIV: Findings From an International Cohort
Source: Liver Int. 2024 Dec 10;45(1):e16203. doi: 10.1111/liv.16203 (PMC11629931; doi:10.1111/liv.16203)
Supplement: Supplementary file 1 — Data S1. [file LIV-45-0-s001.docx]

Supplement to: Harney et al. **Unsuccessful direct-acting antiviral hepatitis C treatment among people with HIV: Findings from a multinational cohort study**

Brendan L. Harney^1,2,3^, Rachel Sacks-Davis^1,2,4^, Daniela K. van Santen^1,5^, Ashleigh C. Stewart^1,2^, Gail V. Matthews^6,7^, Joanne M. Carson^6^, Marina B. Klein^8^, Karine Lacombe^9,10^, Linda Wittkop^11,12,13^, Dominque Salmon^14^, Olivier Leleux^12^, Laurence Merchadou^12^, Marc van der Valk^15,16,17^, Colette Smit^17^, Maria Prins^5,15,16^, Anders Boyd^5,16,17^, Juan Berenguer^18,19,20^, Inmaculada Jarrin^18,19,20^, Andri Rauch^21^, Margaret E. Hellard*^1,2,3,22^, Joseph S. Doyle*^1,2,3^; on behalf of The International Collaboration on Hepatitis C Elimination in HIV Cohorts (InCHEHC). *Joint senior authors

1 Disease Elimination Program, Burnet Institute, Melbourne, Australia

2 School of Public Health and Preventive Medicine, Monash University, Melbourne, Australia

3 Department of Infectious Diseases, Alfred Health and Monash University, Melbourne, Australia

4 School of Population and Global Health, University of Melbourne, Melbourne, Australia

5 Department of Infectious Diseases, Research and Prevention, Public Health Service of Amsterdam, Amsterdam, The Netherlands

6 The Kirby Institute, UNSW, Sydney, Australia

7 St Vincent’s Hospital, Sydney, Australia

8 Division of Infectious Diseases and Chronic Viral Illness Service, Department of Medicine, McGill University Health Centre, Montreal, Canada

9 Sorbonne Université, INSERM, Institut Pierre Louis d’Épidémiologie et de Santé Publique, Paris, France

10 Service de Maladies Infectieuses, Hôpital Saint-Antoine, APHP, Paris, France

11 CHU Bordeaux, Service d’information médicale, F-33000, Bordeaux, France

12 University of Bordeaux, INSERM, Bordeaux Population Health Research Centre, U1219, CIC-EC 1401, Bordeaux, France

13 INRIA SISTM team, Talence, France

14 Université Paris Descartes, Service Maladies Infectieuses et Tropicales, AP-HP, Hôpital Cochin, Paris, France

15 Department of Infectious Diseases, Amsterdam University Medical Centers, University of Amsterdam, Amsterdam, Netherlands.

16 Amsterdam Infection & Immunity Institute, Amsterdam University Medical Centers, University of Amsterdam, Amsterdam, Netherlands

17 Stichting HIV Monitoring, Amsterdam, The Netherlands

18 Centro de Investigación Biomédica en Red de Enfermedades Infecciosas (CIBERINFEC), Madrid, Spain

19 Infectious Diseases, Hospital General Universitario Gregorio Marañón (IsSGM), Madrid, Spain

20 Instituto de Salud Carlos III, Madrid, Spain

21 Department of Infectious Diseases, Inselspital, Bern University Hospital, University of Bern, Bern, Switzerland

22 Doherty Institute and School of Population and Global Health, University of Melbourne, Melbourne, Australia

Contents

[InCHEHC study group 3](#_Toc181095890)

[Supplementary Methods 4](#_Toc181095891)

[Covariables at DAA initiation 4](#_Toc181095892)

[Supplementary methods figure 1. Hypothetical scenarios of inclusion in no SVR4+ test analysis and unsuccessful treatment analysis 6](#_Toc181095893)

[Supplementary results 7](#_Toc181095894)

[Supplementary results table 1. Demographic, behavioural, and clinical characteristics overall and by SVR4+ testing 7](#_Toc181095895)

[Supplementary results table 2. Covariables among people with complete and incomplete data for analysis of unsuccessful treatment 10](#_Toc181095896)

[Supplementary results table 3. Unsuccessful treatment with covariables for sex at birth and GBM in place of population group 12](#_Toc181095897)

[Supplementary results table 4. Unsuccessful treatment with covariable for HCV DAA treatment type included 14](#_Toc181095898)

[Supplementary results table 5. Unsuccessful treatment with covariable for transient elastography based on multiple imputation without imputation of the outcome, n=4091 16](#_Toc181095899)

[Supplementary results table 6. Sensitivity analysis of SVR12+ compared to primary analysis of SVR4+ to define unsuccessful treatment 18](#_Toc181095900)

[Supplementary results table 7. Multiple imputation diagnostics of observed, imputed, and completed distributions of covariables for primary outcome of unsuccessful treatment and secondary outcome of no SVR4+ test 16](#_Toc181095901)

[Supplementary results table 8. Unsuccessful treatment without outcome imputed, with outcome imputed and complete case analysis 18](#_Toc181095902)

[Supplementary results table 9. Unsuccessful treatment based on recent injection drug use 20](#_Toc181095903)

[Supplementary results table 10. SVR4+ testing complete case analysis compared to multiple imputation 22](#_Toc181095904)

[Supplementary results figure 1. Restricted cubic spline of CD4 cell count with reference of 500 cells/mm^3^ 24](#_Toc181095905)

[Supplementary results figure 2. Restricted cubic spline of age with reference of 50 years 25](#_Toc181095906)

[Supplementary results figure 3. Restricted cubic spline of years since HIV diagnosis with reference of 15 years 26](#_Toc181095907)

[Supplementary results figure 4. Restricted cubic spline of years since HCV diagnosis with reference of seven years 27](#_Toc181095908)

[References 28](#_Toc181095909)

[InCHEHC acknowledgments - all cohorts 29](#_Toc181095910)

## InCHEHC study group

**Coordinating centre, ACCESS and Co-EC**: Margaret Hellard, Rachel Sacks-Davis, Daniela van Santen, Ashleigh Stewart, Tianhui Ke, Yanqin Zhang, **ACCESS**: Mark Stoove, Rebecca Guy, Alisa Pedrana, Jason Asselin, Joshua Dawe, Anna Wilkinson **ATHENA**: Anders Boyd, Colette Smit, Marc van der Valk, Janke Schinkel, **ANRS CO13 HEPAVIH**: Linda Wittkop, Dominique Salmon, Philippe Sogni, Laure Esterle, Camille Gilbert, Laurence Merchadou, Stephanie Gillet, Coralie Khan, **ANRS CO3 AQUITAINE**: Fabrice Bonnet, Linda Wittkop, Olivier Leleux, Fabien Le Marec, Adelaïde Perrier **CEASE**: Gail Matthews, Ineke Shaw, Marianne Martinello, Tanya Applegate, Joanne Carson; **co-EC**: Joseph Doyle, Brendan Harney, Melissa Bryant, **CoRIS**: Inmaculada Jarrín Vera, Juan Berenguer, Belén Alejos, Jeffrey V Lazarus, Cristina Moreno, Rebeca Izquierdo, Marta Rava, **CCC**: Marina Klein, Shouao Wang, Jessica Lumia, Costa Pexos, Hansi Peiris, Sahar Saeed, Erica Moodie, Jim Young, Neora Pick, Brian Conway, Mark Hull, Alex Wong, John Gill, Lisa Barrett, Jeff Cohen, Joseph Cox, Pierre Cote, Shariq Haider, Danielle Rouleau, Marie-Louise Vachon, Anita Rachlis, Roger Sandre, Sharon Walmsley, Aida Sadr, Curtis Cooper, Steve Sanche, **SHCS**: Andri Rauch, Catrina Mugglin, Luisa Salazar-Viscaya, Katharina Kusejko, **MOSAIC**: Maria Prins, Kris Hage, **SAIDCC**: Karine Lacombe, Maria-Bernarda Requena, Pierre-Marie Girard, Matthieu Brucker, Jean-Paul Vincensini.

**Cohort name abbreviations**: ACCESS: Australian Collaboration for Coordinated Enhanced Sentinel Surveillance. ATHENA: AIDS Therapy Evaluation in the Netherlands. AQUITAINE: ANRS CO3 AQUITAINE/AquiVIH-NA – prospective clinical based HIV cohort. CCC: Canadian Co-infection Cohort. CEASE: Control and Elimination within AuStralia of Hepatitis C from people living with HIV. Co-EC: Eliminating hepatitis C transmission by enhancing care and treatment among HIV co-infected individuals. CORIS: The cohort of Spanish HIV research network. HEPAVIH: Clinical Centres Collaborations of Subjects Co-infected with HIV and HCV. MOSAIC: MSM Observational Study of Acute Infection with hepatitis C. SAIDCC: Saint-Antoine Infectious Disease Clinical Cohort. SHCS: Swiss HIV cohort Study.

## Supplementary Methods

### Covariables at DAA initiation

Key population group

We defined key population groups as: gay and bisexual males, males with a history of injecting drug use, females with a history of injecting drug use, males with heterosexual or other exposure and females with heterosexual or other exposure. Gay and bisexual males were classified on the basis of having gender recorded as male and either HIV and/or HCV exposure being recorded as sexual transmission with same-sex partner/s, or sexuality being recorded as gay or bisexual. History of injection drug use was derived from HIV/HCV exposure. People who had both GBM and injection drug use recorded were defined as GBM due to injecting among GBM predominantly occurring in the context of sexualised drug use among GBM (1). Heterosexual or other exposure included all other and unknown exposures to HIV and HCV.

HIV viral load

HIV viral load was included as a binary variable indicating viral suppression (HIV viral load <200 copies/mL) (2).

CD4+ cell count

CD4+ cell count was included as a categorical variable of <200 cells/mm^3^, 200 to 350 cells/mm^3^, 350 to 499 cells/mm^3^ and ≥500 cells/mm^3^.

HCV genotype

HCV genotype data were examined as a categorical variable including Genotypes 1, 2, 3 and 4. Due to their rarity and all people being successfully treated, genotypes 5 and 6 were excluded from regression analyses.

Previous interferon treatment

Previous interferon-based treatment was defined on the basis of a previous treatment being recorded within the dataset; the lack of previous treatment was not recorded and ‘no’ reflects people without these data recorded.

Possible cirrhosis

Possible cirrhosis was defined as a transient elastography score > 12.5kPa or FIB-4 > 3.25 depending upon data availability. Where people had both recorded, the one which was performed closest to treatment commencement date was used.

Transient Elastography

As transient elastography is recognised as a more accurate predictor of cirrhosis than serum biomarkers (3), we used this as an alternative marker of cirrhosis in categories of <12.5kPa, 12.5-21kPa and greater than 21kPa. We included the latter category as a marker of possible decompensated cirrhosis and related complications of liver disease(4).

Recent injecting drug use

Time updated data on recent injecting drug use varied across cohorts and was collected for all but two of the nine cohorts. Recall periods ranged from one month to six months or ‘since last follow-up visit’. We defined recent injecting drug use (yes/no) based on responses within 12 months prior to the treatment date.

### Supplementary methods figure 1. Hypothetical scenarios of inclusion in no SVR4+ test analysis and unsuccessful treatment analysis

| **Example** | **Pre-Treatment** | **During or at end of treatment** | **SVR4** | **SVR12** | **SVR24** | **SVR48** |
| --- | --- | --- | --- | --- | --- | --- |
| **A** | **RNA+** | **RNA-** | **RNA-** | **RNA-** | **RNA-** | **RNA-** |
| **B** | **RNA+** | **-** | **-** | **-** | **-** |  |
| **C** | **RNA+** | **RNA-** | **RNA+** | **-** | **-** | **-** |
| **D** | **RNA+** | **RNA+** | **RNA+** | **RNA-** | **-** | **-** |
| **E** | **RNA+** | **-** | **-** | **-** | **-** | **RNA+** |
| **F** | **RNA+** | **-** | **-** | **-** | **-** | **RNA-** |
| **G** | **RNA+** | **-** | **RNA-** | **RNA+** | **-** | **-** |

Example A shows a “best case scenario” whereby someone is treated and is HCV RNA negative at all subsequent visits. They would be classified as having an SVR test in the no SVR4+ test analysis and being cured in the unsuccessful treatment analysis.

Example B shows someone who starts treatment but does not have any follow-up records of HCV RNA testing; this person would be classified as having no SVR test in the no SVR4+ test analysis and not eligible for the unsuccessful treatment analysis.

Example C shows someone who is HCV RNA positive at SVR4; they would be included in the no SVR4+ test analysis as having an SVR test. This person would be classified as unsuccessfully treated. They would be considered unsuccessfully treated due to relapse.

Example D shows someone who is HCV RNA positive at SVR4; they would be included in the no SVR4+ test analysis as having an SVR test. This person would be classified as unsuccessfully treated.

Example E shows someone who starts treatment but does not have any HCV RNA tests until SVR48, i.e., almost a year later; this person would be included in the no SVR4+ test analysis as having a SVR test as this is their first HCV RNA test 4 or more weeks after their estimated end of treatment. They would be classified as unsuccessfully treated. They would be considered unsuccessfully treated due to non-response.

Example F is similar to example E but classified as successfully treated.

Example G shows someone who is HCV RNA negative at SVR4; they would be included in the SVR4+ analysis has having an SVR test and as successfully treated.

## Supplementary results

### Supplementary results table 1. Demographic, behavioural, and clinical characteristics overall and by SVR4+ testing

|  | Overall  (N=4468) | No SVR4+ test (n=370) | SVR4+ test  (n=4098) |
| --- | --- | --- | --- |
| Age, mean (SD, Range)^a^ | 50 (8.9, 21-85) | 49 (9.2, 25-74) | 50 (8.9, 21-85) |
| **Country** |  |  |  |
| Australia | 462 (10.3) | 23 (5.0) | 439 (95.0) |
| Canada | 703 (15.7) | 48 (6.8) | 655 (93.2) |
| France | 880 (19.7) | 65 (7.4) | 815 (92.6) |
| The Netherlands | 1,026 (23.0) | 47 (4.6) | 979 (95.4) |
| Spain | 585 (13.1) | 116 (19.8) | 469 (80.2) |
| Switzerland | 812 (18.2) | 71 (8.7) | 741 (91.3) |
|  |  |  |  |
| **Population group**^b^ |  |  |  |
| GBM | 1,986 (44.4) | 132 (6.6) | 1,854 (93.4) |
| Male Hx of IDU | 1,145 (25.6) | 112 (9.8) | 1,033 (90.2) |
| Female Hx of IDU | 491 (11.0) | 43 (8.8) | 448 (91.2) |
| Male Hetero/other | 503 (11.3) | 52 (10.3) | 451 (89.7) |
| Female Hetero/other | 333 (7.5) | 31 (9.3) | 302 (90.7) |
| Unknown/missing | 10 (0.2) |  | 10 (100.0) |
|  |  |  |  |
| **Recent injection drug use**^c^ |  |  |  |
| No | 1,556 (34.8) | 118 (7.6) | 1,438 (92.4) |
| Yes | 365 (8.2) | 23 (6.3) | 342 (93.7) |
| Unknown/missing | 2,547 (57.0) | 229 (9.0) | 2,318 (91.0) |
|  |  |  |  |
| **Genotype**^d^ |  |  |  |
| GT1 | 2,533 (56.7) | 192 (7.6) | 2,341 (92.4) |
| GT2 | 126 (2.8) | 11 (8.7) | 115 (91.3) |
| GT3 | 657 (14.7) | 60 (9.1) | 597 (90.9) |
| GT4 | 673 (15.1) | 55 (8.2) | 618 (91.8) |
| GT5 | 1 (0.0) | 0 | 1 (100.0) |
| GT6 | 6 (0.1) | 0 | 6 (100.0) |
| Unknown/missing | 472 (10.6) | 52 (11.0) | 420 (89.0) |
|  |  |  |  |
| **CD4+ cell coun**t^d^ |  |  |  |
| 500+ | 2,726 (61.0) | 218 (8.0) | 2,508 (92.0) |
| 499-350 | 757 (16.9) | 56 (7.4) | 701 (92.6) |
| 200-349 | 518 (11.6) | 52 (10.0) | 466 (90.0) |
| <200 | 247 (5.5) | 29 (11.7) | 218 (88.3) |
| Unknown/missing | 220 (4.9) | 15 (6.8) | 205 (93.2) |
|  |  |  |  |
| **Transient elastography score**^c,d^ | |  |  |
| <12.5 | 2,544 (56.9) | 191 (7.5) | 2,353 (92.5) |
| 12.5-21 | 302 (6.8) | 16 (5.3) | 286 (94.7) |
| ≥21 | 301 (6.7) | 31 (10.3) | 270 (89.7) |
| Unknown/missing | 1,321 (29.6) | 132 (10.0) | 1,189 (90.0) |
|  |  |  |  |
| **FIB-4 Score**^c,d^ |  |  |  |
| ≤3.25 | 2,864 (64.1) | 246 (8.6) | 2,618 (91.4) |
| >3.25 | 546 (12.2) | 53 (9.7) | 493 (90.3) |
| Unknown/missing | 1,058 (23.7) | 71 (6.7) | 987 (93.3) |
|  |  |  |  |
| **Possible cirrhosis**^e^ |  |  |  |
| No | 3,467 (77.6) | 281 (8.1) | 3,186 (91.9) |
| Yes | 780 (17.5) | 66 (8.5) | 714 (91.5) |
| Unknown/missing | 221 (4.9) | 23 (10.4) | 198 (89.6) |
|  |  |  |  |
| **Years since HIV diagnosis** |  |  |  |
| <10 | 1,346 (30.1) | 125 (9.3) | 1,221 (90.7) |
| 10-19 | 1,429 (32.0) | 130 (9.1) | 1,299 (90.9) |
| 20-29 | 1,293 (28.9) | 77 (6.0) | 1,216 (94.0) |
| 30+ | 357 (8.0) | 36 (10.1) | 321 (89.9) |
| Unknown/missing | 43 (1.0) | 2 (4.7) | 41 (95.3) |
|  |  |  |  |
| **Years since first HCV positive test** | |  |  |
| <10 | 2,663 (59.6) | 223 (8.4) | 2,440 (91.6) |
| 10-19 | 1,231 (27.6) | 102 (8.3) | 1,129 (91.7) |
| 20+ | 518 (11.6) | 40 (7.7) | 478 (92.3) |
| Unknown/missing | 56 (1.3) | 5 (8.9) | 51 (91.1) |
|  |  |  |  |
| **HIV RNA viral load**^d^ |  |  |  |
| <200 copies/ml | 4,066 (91.0) | 326 (8.0) | 3,740 (92.0) |
| ≥200 copies/ml | 203 (4.5) | 33 (16.3) | 170 (83.7) |
| Unknown/missing | 199 (4.5) | 11 (5.5) | 188 (94.5) |
|  |  |  |  |
| **Previous HCV treatmen**t^f^ |  |  |  |
| No | 3,316 (74.2) | 310 (9.3) | 3,006 (90.7) |
| Yes | 1,152 (25.8) | 60 (5.2) | 1,092 (94.8) |
|  |  |  |  |
| **DAA prescription** |  |  |  |
| Sofosbuvir | 179 (4.0) | 11 (6.1) | 168 (93.9) |
| Sofosbuvir/Ledipasvir | 1,934 (43.3) | 123 (6.4) | 1,811 (93.6) |
| Sofosbuvir/Velpatasvir | 577 (12.9) | 111 (19.2) | 466 (80.8) |
| Elbasvir/Grasoprevir | 330 (7.4) | 27 (8.2) | 303 (91.8) |
| Sofosbuvir/Daclatasvir | 685 (15.3) | 38 (5.5) | 647 (94.5) |
| Paritaprevir/Ombitasvir/Ritonavir ± Dasabuvir | 453 (10.1) | 23 (5.1) | 430 (94.9) |
| Glecaprevir/Pibrentasvir | 156 (3.5) | 29 (18.6) | 127 (81.4) |
| Sofosbuvir/Velpatasvir/Voxilaprevir | 6 (0.1) | 1 (16.7) | 5 (83.3) |
| Sofosbuvir/Simeprevir | 148 (3.3) | 7 (4.7) | 141 (95.3) |

Missing data were uncommon for most variables, except for those known to not be collected by all cohorts (transient elastography and recent injection drug use); most other variables had <5% missing data with an exception for genotype (10.6% missing). Based on the primary outcome of unsuccessful treatment, of the 4098 people eligible for inclusion in this analysis, 83.2% had all data available; there was minimal difference in this among people who unsuccessfully treated (81.6%) and those successfully treated (83.3%). There was some variation in the distribution of covariables including CD4+ cell count and HIV viral load among people who were defined as complete cases and those who were not (Supplementary results table 1).

### Supplementary results table 2. Covariables among people with complete and incomplete data for analysis of unsuccessful treatment

|  | Incomplete Cases, n=736 | Complete Cases, n=3362 |
| --- | --- | --- |
| Age (10 years) |  |  |
| 20-29 | 16 (19.8) | 65 (80.2) |
| 30-39 | 79 (16.3) | 406 (83.7) |
| 40-49 | 214 (17.4) | 1,014 (82.6) |
| 50-59 | 300 (16.4) | 1,530 (83.6) |
| 60-69 | 63 (14.7) | 365 (85.3) |
| 70-79 | 7 (19.4) | 29 (80.6) |
|  |  |  |
| Population group |  |  |
| GBM | 314 (16.9) | 1,540 (83.1) |
| Male Hx of IDU | 168 (16.3) | 865 (83.7) |
| Female Hx of IDU | 56 (12.5) | 392 (87.5) |
| Male Hetero/other | 89 (19.7) | 362 (80.3) |
| Female Hetero/other | 52 (17.2) | 250 (82.8) |
|  |  |  |
| Previous HCV treatment |  |  |
| No | 550 (18.3) | 2,456 (81.7) |
| Yes | 139 (12.7) | 953 (87.3) |
|  |  |  |
| CD4+ cell count |  |  |
| 500+ | 317 (12.6) | 2,191 (87.4) |
| 499-350 | 77 (11.0) | 624 (89.0) |
| 200-349 | 53 (11.4) | 413 (88.6) |
| <200 | 37 (17.0) | 181 (83.0) |
|  |  |  |
| Possible cirrhosis |  |  |
| No | 420 (13.2) | 2,766 (86.8) |
| Yes | 71 (9.9) | 643 (90.1) |
|  |  |  |
| Genotype |  |  |
| GT1 | 173 (7.4) | 2,167 (92.6) |
| GT2 | 11 (9.6) | 104 (90.4) |
| GT3 | 52 (8.7) | 545 (91.3) |
| GT4 | 33 (5.3) | 586 (94.7) |
| GT5 | 0 | 1 (100.0) |
| GT6 | 0 | 6 (100.0) |
|  |  |  |
| Years since HIV diagnosis |  |  |
| <10 | 223 (18.3) | 998 (81.7) |
| 10-19 | 195 (15.0) | 1,104 (85.0) |
| 20-29 | 188 (15.5) | 1,028 (84.5) |
| 30+ | 42 (13.1) | 279 (86.9) |
|  |  |  |
| Years since first HCV positive test | |  |
| <10 | 473 (19.4) | 1,967 (80.6) |
| 10-19 | 104 (9.2) | 1,025 (90.8) |
| 20+ | 61 (12.8) | 417 (87.2) |
|  |  |  |
| HIV RNA viral load |  |  |
| <200 copies/ml | 469 (12.5) | 3,271 (87.5) |
| ≥200 copies/ml | 32 (18.8) | 138 (81.2) |

### Supplementary results table 3. Unsuccessful treatment with covariables for sex at birth and GBM in place of population group

|  | Without outcome imputed, n=4081 | With outcome  imputed, n=4451 | Complete case  analysis, n=3355 |
| --- | --- | --- | --- |
|  | aOR (95% CI) | aOR (95% CI) | aOR (95% CI) |
| Age (10 years) | 1.00 (0.85-1.19) | 1.01 (0.85-1.20) | 1.04 (0.86-1.26) |
|  |  |  |  |
| Sex at birth |  |  |  |
| Male | 1 | 1 | 1 |
| Female | 0.58 (0.38-0.89) | 0.60 (0.38-0.93) | 0.58 (0.36-0.94) |
|  |  |  |  |
| Gay or bisexual male |  |  |  |
| Yes | 1 | 1 | 1 |
| No | 1.39 (0.98-1.98) | 1.36 (0.95-1.94) | 1.29 (0.86-1.93) |
|  |  |  |  |
| Previous HCV treatment |  |  |  |
| No | 1 | 1 | 1 |
| Yes | 1.22 (0.87-1.72) | 1.24 (0.89-1.73) | 1.15 (0.80-1.65) |
|  |  |  |  |
| CD4+ cell count |  |  |  |
| 500+ | 1 | 1 | 1 |
| 499-350 | 1.23 (0.82-1.83) | 1.26 (0.86-1.84) | 1.27 (0.84-1.94) |
| 200-349 | 1.93 (1.29-2.88) | 1.87 (1.25-2.78) | 1.78 (1.14-2.77) |
| <200 | 1.81 (0.99-3.30) | 1.92 (1.06-3.50) | 2.11 (1.15-3.86) |
|  |  |  |  |
| Possible cirrhosis |  |  |  |
| No | 1 | 1 | 1 |
| Yes | 1.27 (0.88-1.82) | 1.23 (0.85-1.79) | 1.14 (0.77-1.69) |
|  |  |  |  |
| Genotype |  |  |  |
| GT1 | 1 | 1 | 1 |
| GT2 | 1.93 (0.91-4.10) | 1.83 (0.90-3.74) | 1.70 (0.76-3.82) |
| GT3 | 1.19 (0.78-1.81) | 1.16 (0.77-1.75) | 1.12 (0.72-1.74) |
| GT4 | 1.66 (1.12-2.46) | 1.70 (1.15-2.51) | 1.58 (1.05-2.37) |
|  |  |  |  |
| Years since HIV diagnosis |  |  |  |
| <10 | 1 | 1 | 1 |
| 10-19 | 0.90 (0.60-1.35) | 0.86 (0.56-1.32) | 1.12 (0.71-1.77) |
| 20-29 | 0.68 (0.42-1.10) | 0.65 (0.39-1.07) | 0.90 (0.53-1.52) |
| 30+ | 0.63 (0.32-1.27) | 0.62 (0.30-1.27) | 0.64 (0.29-1.42) |
|  |  |  |  |
| Years since first HCV positive test |  |  |  |
| <10 | 1 | 1 | 1 |
| 10-19 | 1.08 (0.71-1.62) | 1.10 (0.72-1.67) | 1.11 (0.72-1.73) |
| 20+ | 1.15 (0.64-2.07) | 1.19 (0.66-2.14) | 1.15 (0.61-2.17) |
|  |  |  |  |
| HIV RNA viral load |  |  |  |
| <200 copies/ml | 1 | 1 | 1 |
| ≥200 copies/ml | 1.13 (0.59-2.17) | 1.13 (0.61-2.11) | 1.19 (0.58-2.43) |

### Supplementary results table 4. Unsuccessful treatment with covariable for HCV DAA treatment type included

|  | Without outcome imputed, n=4081 | | With outcome imputed, n=4451 | | Complete case analysis, n=3355 |
| --- | --- | --- | --- | --- | --- |
|  | aOR (95% CI) |  | aOR (95% CI) |  | aOR (95% CI) |
| Age (10 years) | 1.00 (0.85-1.19) |  | 1.01 (0.85-1.20) |  | 1.05 (0.86-1.27) |
|  |  |  |  |  |  |
| Population group |  |  |  |  |  |
| GBM | 1 |  | 1 |  | 1 |
| Male Hx of IDU | 1.46 (0.98-2.17) |  | 1.42 (0.95-2.13) |  | 1.45 (0.95-2.21) |
| Female Hx of IDU | 0.60 (0.32-1.15) |  | 0.62 (0.33-1.17) |  | 0.62 (0.32-1.22) |
| Male Hetero/other | 1.46 (0.92-2.32) |  | 1.45 (0.92-2.28) |  | 1.14 (0.66-1.98) |
| Female Hetero/other | 1.17 (0.66-2.08) |  | 1.15 (0.63-2.08) |  | 0.99 (0.51-1.93) |
|  |  |  |  |  |  |
| Previous HCV treatment |  |  |  |  |  |
| No | 1 |  | 1 |  | 1 |
| Yes | 1.23 (0.87-1.74) |  | 1.23 (0.87-1.75) |  | 1.18 (0.81-1.72) |
|  |  |  |  |  |  |
| CD4+ cell count |  |  |  |  |  |
| 500+ | 1 |  | 1 |  | 1 |
| 499-350 | 1.22 (0.82-1.81) |  | 1.21 (0.79-1.83) |  | 1.25 (0.82-1.91) |
| 200-349 | 1.93 (1.29-2.88) |  | 1.98 (1.35-2.92) |  | 1.75 (1.12-2.73) |
| <200 | 1.72 (0.96-3.08) |  | 1.74 (0.99-3.04) |  | 2.06 (1.12-3.77) |
|  |  |  |  |  |  |
| Possible cirrhosis |  |  |  |  |  |
| No | 1 |  | 1 |  | 1 |
| Yes | 1.24 (0.84-1.83) |  | 1.21 (0.83-1.77) |  | 1.09 (0.73-1.64) |
|  |  |  |  |  |  |
| Genotype |  |  |  |  |  |
| GT1 | 1 |  | 1 |  | 1 |
| GT2 | 0.99 (0.44-2.23) |  | 1.04 (0.46-2.34) |  | 0.88 (0.35-2.21) |
| GT3 | 1.02 (0.63-1.64) |  | 1.05 (0.67-1.66) |  | 0.95 (0.57-1.59) |
| GT4 | 1.38 (0.91-2.09) |  | 1.57 (1.05-2.35) |  | 1.32 (0.87-2.01) |
|  |  |  |  |  |  |
| Years since HIV diagnosis |  |  |  |  |  |
| <10 | 1 |  | 1 |  | 1 |
| 10-19 | 0.91 (0.60-1.37) |  | 0.90 (0.60-1.35) |  | 1.12 (0.71-1.76) |
| 20-29 | 0.72 (0.44-1.18) |  | 0.74 (0.45-1.20) |  | 0.90 (0.53-1.53) |
| 30+ | 0.68 (0.34-1.36) |  | 0.69 (0.35-1.36) |  | 0.68 (0.31-1.51) |
|  |  |  |  |  |  |
| Years since first HCV positive test |  |  |  |  |  |
| <10 | 1 |  | 1 |  | 1 |
| 10-19 | 1.07 (0.70-1.64) |  | 1.09 (0.71-1.67) |  | 1.10 (0.71-1.68) |
| 20+ | 1.11 (0.60-2.04) |  | 1.09 (0.58-2.06) |  | 1.08 (0.58-2.02) |
|  |  |  |  |  |  |
| HIV RNA viral load |  |  |  |  |  |
| <200 copies/ml | 1 |  | 1 |  | 1 |
| ≥200 copies/ml | 1.13 (0.58-2.21) |  | 1.13 (0.57-2.21) |  | 1.19 (0.58-2.43) |
|  |  |  |  |  |  |
| DAA prescription |  |  |  |  |  |
| Sofosbuvir/Ledipasvir | 1 |  | 1 |  | 1 |
| Sofosbuvir | 3.35 (1.83-6.14) |  | 3.12 (1.69-5.74) |  | 3.28 (1.62-6.64) |
| Sofosbuvir/Velpatasvir | 1.45 (0.88-2.37) |  | 1.33 (0.82-2.16) |  | 1.37 (0.78-2.39) |
| Elbasvir/Grasoprevir | 1.88 (1.11-3.20) |  | 1.78 (1.04-3.04) |  | 1.91 (1.10-3.31) |
| Paritaprevir/Ombitasvir/Ritonavir ± Dasabuvir | 0.82 (0.47-1.46) |  | 0.81 (0.46-1.44) |  | 0.85 (0.46-1.57) |
| Daclatasvir/Sofosbuvir | 1.06 (0.64-1.76) |  | 1.07 (0.65-1.76) |  | 1.05 (0.61-1.81) |
| Glecaprevir/Pibrentasvir | 1.25 (0.53-3.00) |  | 1.14 (0.48-2.72) |  | 1.23 (0.48-3.18) |
| Sofosbuvir/Simeprevir | 1.16 (0.54-2.50) |  | 1.09 (0.51-2.36) |  | 1.09 (0.45-2.63) |

### Supplementary results table 5. Unsuccessful treatment with covariable for transient elastography based on multiple imputation without imputation of the outcome, n=4091

|  |  |  | With CD4+ cell count |  | Without CD+ cell count | |
| --- | --- | --- | --- | --- | --- | --- |
|  | OR (95% CI) |  | aOR (95% CI) | | aOR (95% CI) | |
| Age (10 years) | 1.03 (0.88-1.20) |  | 1.01 (0.85-1.20) | | 1.02 (0.86-1.20) | |
|  |  |  |  |  |  |  |
| Population group |  |  |  |  |  |  |
| GBM | 1 |  | 1 |  | 1 |  |
| Male Hx of IDU | 1.44 (1.01-2.06) |  | 1.35 (0.91-2.01) | | 1.38 (0.93-2.06) | |
| Female Hx of IDU | 0.60 (0.33-1.11) |  | 0.59 (0.31-1.11) | | 0.60 (0.32-1.14) | |
| Male Hetero/other | 1.52 (0.97-2.36) |  | 1.37 (0.86-2.17) | | 1.42 (0.90-2.26) | |
| Female Hetero/other | 1.25 (0.72-2.17) |  | 1.11 (0.63-1.97) | | 1.16 (0.66-2.05) | |
|  |  |  |  |  |  |  |
| Previous HCV treatment |  |  |  |  |  |  |
| No | 1 |  | 1 |  | 1 |  |
| Yes | 1.21 (0.88-1.67) |  | 1.21 (0.86-1.71) | | 1.16 (0.83-1.63) | |
|  |  |  |  |  |  |  |
| CD4+ cell count |  |  |  |  |  |  |
| 500+ | 1 |  | 1 |  |  |  |
| 499-350 | 1.24 (0.83-1.84) |  | 1.22 (0.82-1.82) | |  |  |
| 200-349 | 2.05 (1.39-3.05) |  | 1.98 (1.32-2.97) | |  |  |
| <200 | 1.89 (1.09-3.28) |  | 1.86 (1.05-3.29) | |  |  |
|  |  |  |  |  |  |  |
| Transient elastography |  |  |  |  |  |  |
| score |  |  |  |  |  |  |
| <12.5 | 1 |  | 1 |  | 1 |  |
| 12.5-21 | 1.28 (0.74-2.20) |  | 1.18 (0.67-2.08) | | 1.36 (0.80-2.31) | |
| ≥21 | 1.53 (0.92-2.55) |  | 1.31 (0.76-2.25) | | 1.49 (0.88-2.52) | |
|  |  |  |  |  |  |  |
| Genotype |  |  |  |  |  |  |
| GT1 | 1 |  | 1 |  | 1 |  |
| GT2 | 1.77 (0.85-3.65) |  | 1.79 (0.85-3.77) | | 1.86 (0.89-3.91) | |
| GT3 | 1.24 (0.83-1.85) |  | 1.15 (0.76-1.74) | | 1.24 (0.82-1.87) | |
| GT4 | 1.44 (0.97-2.14) |  | 1.56 (1.04-2.33) | | 1.49 (1.00-2.22) | |
|  |  |  |  |  |  |  |
| Years since HIV |  |  |  |  |  |  |
| diagnosis |  |  |  |  |  |  |
| <10 | 1 |  | 1 |  | 1 |  |
| 10-19 | 0.99 (0.69-1.41) |  | 0.91 (0.60-1.36) | | 0.90 (0.60-1.35) | |
| 20-29 | 0.85 (0.58-1.25) |  | 0.70 (0.43-1.13) | | 0.71 (0.44-1.15) | |
| 30+ | 0.75 (0.40-1.40) |  | 0.65 (0.32-1.30) | | 0.65 (0.32-1.32) | |
|  |  |  |  |  |  |  |
| Years since first HCV |  |  |  |  |  |  |
| positive test |  |  |  |  |  |  |
| <10 | 1 |  | 1 |  | 1 |  |
| 10-19 | 1.10 (0.79-1.54) |  | 1.09 (0.72-1.64) | | 1.09 (0.72-1.65) | |
| 20+ | 1.09 (0.67-1.75) |  | 1.16 (0.65-2.10) | | 1.19 (0.66-2.15) | |
|  |  |  |  |  |  |  |
| HIV RNA viral load |  |  |  |  |  |  |
| <200 copies/ml | 1 |  | 1 |  | 1 |  |
| ≥200 copies/ml | 1.29 (0.67-2.51) |  | 1.11 (0.57-2.19) | | 1.28 (0.68-2.42) | |

### Supplementary results table 6. Sensitivity analysis of SVR12+ compared to primary analysis of SVR4+ to define unsuccessful treatment

|  |  | SVR4+, n=4091^a^ |  | SVR12+ without outcome imputed, n=3928 |  | SVR12+ with outcome imputed, n=4461 |
| --- | --- | --- | --- | --- | --- | --- |
|  |  | aOR (95% CI) |  | aOR (95% CI) |  | aOR (95% CI) |
| Age (10 years) |  | 1.00 (0.85-1.19) |  | 1.00 (0.85-1.19) |  | 1.00 (0.85-1.19) |
|  |  |  |  |  |  |  |
| Population group |  |  |  |  |  |  |
| GBM |  | 1 |  | 1 |  | 1 |
| Male Hx of IDU |  | 1.37 (0.92-2.03) |  | 1.37 (0.92-2.03) |  | 1.31 (0.88-1.94) |
| Female Hx of IDU |  | 0.59 (0.31-1.12) |  | 0.59 (0.31-1.12) |  | 0.57 (0.30-1.08) |
| Male Hetero/other |  | 1.39 (0.88-2.19) |  | 1.38 (0.88-2.19) |  | 1.40 (0.89-2.21) |
| Female Hetero/other |  | 1.11 (0.63-1.97) |  | 1.12 (0.63-1.97) |  | 1.08 (0.62-1.89) |
|  |  |  |  |  |  |  |
| Previous HCV treatment |  |  |  |  |  |  |
| No |  | 1 |  | 1 |  | 1 |
| Yes |  | 1.22 (0.87-1.71) |  | 1.22 (0.87-1.71) |  | 1.21 (0.87-1.69) |
|  |  |  |  |  |  |  |
| CD4+ cell count |  |  |  |  |  |  |
| 500+ |  | 1 |  | 1 |  | 1 |
| 499-350 |  | 1.24 (0.83-1.87) |  | 1.24 (0.82-1.85) |  | 1.21 (0.80-1.85) |
| 200-349 |  | 1.95 (1.30-2.93) |  | 1.95 (1.29-2.95) |  | 1.96 (1.29-2.99) |
| <200 |  | 1.81 (1.00-3.29) |  | 1.77 (0.95-3.30) |  | 1.80 (1.05-3.10) |
|  |  |  |  |  |  |  |
| Possible cirrhosis |  |  |  |  |  |  |
| No |  | 1 |  | 1 |  | 1 |
| Yes |  | 1.27 (0.89-1.83) |  | 1.28 (0.89-1.85) |  | 1.27 (0.88-1.83) |
|  |  |  |  |  |  |  |
| Genotype |  |  |  |  |  |  |
| GT1 |  | 1 |  | 1 |  | 1 |
| GT2 |  | 1.79 (0.88-3.62) |  | 1.79 (0.88-3.63) |  | 1.71 (0.83-3.53) |
| GT3 |  | 1.16 (0.77-1.74) |  | 1.16 (0.77-1.74) |  | 1.18 (0.78-1.79) |
| GT4 |  | 1.56 (1.04-2.32) |  | 1.56 (1.05-2.33) |  | 1.63 (1.08-2.44) |
|  |  |  |  |  |  |  |
| Years since HIV diagnosis |  |  |  |  |  |  |
| <10 |  | 1 |  | 1 |  | 1 |
| 10-19 |  | 0.89 (0.60-1.33) |  | 0.89 (0.60-1.34) |  | 0.88 (0.60-1.31) |
| 20-29 |  | 0.69 (0.42-1.12) |  | 0.69 (0.42-1.12) |  | 0.69 (0.43-1.12) |
| 30+ |  | 0.64 (0.32-1.29) |  | 0.65 (0.32-1.30) |  | 0.64 (0.31-1.31) |
|  |  |  |  |  |  |  |
| Years since first HCV positive test | |  |  |  |  |  |
| <10 |  | 1 |  | 1 |  | 1 |
| 10-19 |  | 1.08 (0.72-1.64) |  | 1.08 (0.72-1.63) |  | 1.11 (0.74-1.67) |
| 20+ |  | 1.16 (0.64-2.11) |  | 1.16 (0.64-2.10) |  | 1.15 (0.64-2.07) |
|  |  |  |  |  |  |  |
| HIV RNA viral load |  |  |  |  |  |  |
| <200 copies/ml |  | 1 |  | 1 |  | 1 |
| ≥200 copies/ml |  | 1.09 (0.56-2.11) |  | 1.08 (0.56-2.07) |  | 1.16 (0.60-2.25) |

a. Multivariable analysis from Table 2 in main manuscript

| Supplementary results table 7. Multiple imputation diagnostics of observed, imputed, and completed distributions of covariables for primary outcome of unsuccessful treatment and secondary outcome of no SVR4+ test | | | | | | | |
| --- | --- | --- | --- | --- | --- | --- | --- |
|  | Unsuccessful treatment |  |  |  | No SVR4+ test |  |  |
|  | Observed | Imputed | Completed |  | Observed | Imputed | Completed |
| Age group |  |  |  |  |  |  |  |
| 20-29 | 0.020 | 0.000 | 0.020 |  | 0.020 | 0.100 | 0.020 |
| 30-39 | 0.119 | 0.100 | 0.119 |  | 0.123 | 0.100 | 0.123 |
| 40-49 | 0.301 | 0.500 | 0.301 |  | 0.302 | 0.500 | 0.303 |
| 50-59 | 0.448 | 0.300 | 0.447 |  | 0.441 | 0.200 | 0.441 |
| 60-69 | 0.104 | 0.100 | 0.104 |  | 0.104 | 0.100 | 0.104 |
| 70-79 | 0.009 | 0.000 | 0.009 |  | 0.009 | 0.000 | 0.009 |
|  |  |  |  |  |  |  |  |
| Population group |  |  |  |  |  |  |  |
| GBM | 0.454 | 0.400 | 0.454 |  | 0.446 | 0.400 | 0.446 |
| Male Hx of IDU | 0.253 | 0.100 | 0.253 |  | 0.257 | 0.200 | 0.257 |
| Female Hx of IDU | 0.110 | 0.200 | 0.110 |  | 0.110 | 0.100 | 0.110 |
| Male Hetero/other | 0.110 | 0.300 | 0.111 |  | 0.113 | 0.300 | 0.113 |
| Female Hetero/other | 0.074 | 0.000 | 0.073 |  | 0.074 | 0.000 | 0.074 |
|  |  |  |  |  |  |  |  |
| CD4+ cell count |  |  |  |  |  |  |  |
| 500+ | 0.644 | 0.659 | 0.645 |  | 0.642 | 0.586 | 0.639 |
| 499-350 | 0.180 | 0.127 | 0.177 |  | 0.178 | 0.191 | 0.179 |
| 200-349 | 0.120 | 0.146 | 0.121 |  | 0.122 | 0.150 | 0.123 |
| <200 | 0.056 | 0.068 | 0.057 |  | 0.058 | 0.073 | 0.059 |
|  |  |  |  |  |  |  |  |
| Genotype |  |  |  |  |  |  |  |
| GT1 | 0.638 | 0.629 | 0.637 |  | 0.635 | 0.614 | 0.633 |
| GT2 | 0.032 | 0.019 | 0.030 |  | 0.032 | 0.038 | 0.033 |
| GT3 | 0.162 | 0.181 | 0.164 |  | 0.164 | 0.184 | 0.167 |
| GT4 | 0.168 | 0.171 | 0.169 |  | 0.169 | 0.163 | 0.168 |
|  |  |  |  |  |  |  |  |
| HIV RNA viral |  |  |  |  |  |  |  |
| load |  |  |  |  |  |  |  |
| <200 copies/ml | 0.956 | 0.926 | 0.955 |  | 0.952 | 0.915 | 0.951 |
| ≥200 copies/ | 0.044 | 0.074 | 0.045 |  | 0.048 | 0.085 | 0.049 |
|  |  |  |  |  |  |  |  |
| Years since first HCV positive test | |  |  |  |  |  |  |
| <10 | 0.603 | 0.725 | 0.604 |  | 0.603 | 0.661 | 0.604 |
| 10 to 19 | 0.279 | 0.275 | 0.279 |  | 0.279 | 0.268 | 0.279 |
| 20+ | 0.118 | 0.000 | 0.117 |  | 0.117 | 0.071 | 0.117 |
|  |  |  |  |  |  |  |  |
| Years since HIV diagnosis | |  |  |  |  |  |  |
| <10 | 0.301 | 0.415 | 0.302 |  | 0.304 | 0.302 | 0.304 |
| 10 to 19 | 0.320 | 0.317 | 0.320 |  | 0.323 | 0.395 | 0.323 |
| 20 to 29 | 0.300 | 0.244 | 0.300 |  | 0.293 | 0.256 | 0.292 |
| 30+ | 0.079 | 0.024 | 0.078 |  | 0.081 | 0.047 | 0.080 |
|  |  |  |  |  |  |  |  |
| Possible cirrhosis |  |  |  |  |  |  |  |
| No | 0.817 | 0.778 | 0.815 |  | 0.816 | 0.824 | 0.817 |
| Yes | 0.183 | 0.222 | 0.185 |  | 0.184 | 0.176 | 0.183 |

### Supplementary results table 8. Unsuccessful treatment without outcome imputed, with outcome imputed and complete case analysis

|  |  | SVR4+ without outcome imputed |  | SVR4+ with outcome imputed |  | SVR4+ complete case analysis | |
| --- | --- | --- | --- | --- | --- | --- | --- |
|  |  | aOR (95% CI) |  | aOR (95% CI) |  | aOR (95% CI) |  |
| Age (10 years) |  | 1.00 (0.85-1.19) |  | 1.01 (0.85-1.19) |  | 1.03 (0.85-1.25) |  |
|  |  |  |  |  |  |  |  |
| Population group |  |  |  |  |  |  |  |
| GBM |  | 1 |  | 1 |  | 1 |  |
| Male Hx of IDU |  | 1.37 (0.92-2.03) |  | 1.37 (0.92-2.02) |  | 1.43 (0.92-2.21) |  |
| Female Hx of IDU |  | 0.59 (0.31-1.12) |  | 0.59 (0.31-1.12) |  | 0.63 (0.32-1.26) |  |
| Male Hetero/other |  | 1.39 (0.88-2.19) |  | 1.38 (0.87-2.18) |  | 1.16 (0.67-2.00) |  |
| Female Hetero/other |  | 1.11 (0.63-1.97) |  | 1.11 (0.63-1.97) |  | 0.95 (0.49-1.86) |  |
|  |  |  |  |  |  |  |  |
| Previous HCV treatment |  |  |  |  |  |  |  |
| No |  | 1 |  | 1 |  | 1 |  |
| Yes |  | 1.22 (0.87-1.71) |  | 1.21 (0.86-1.70) |  | 1.10 (0.76-1.58) |  |
|  |  |  |  |  |  |  |  |
| CD4+ cell count |  |  |  |  |  |  |  |
| 500+ |  | 1 |  | 1 |  | 1 |  |
| 499-350 |  | 1.24 (0.83-1.87) |  | 1.23 (0.82-1.83) |  | 1.29 (0.85-1.95) |  |
| 200-349 |  | 1.95 (1.30-2.93) |  | 1.95 (1.30-2.90) |  | 1.87 (1.21-2.89) |  |
| <200 |  | 1.81 (1.00-3.29) |  | 1.79 (1.02-3.15) |  | 2.10 (1.15-3.84) |  |
|  |  |  |  |  |  |  |  |
| Possible cirrhosis |  |  |  |  |  |  |  |
| No |  | 1 |  | 1 |  | 1 |  |
| Yes |  | 1.27 (0.89-1.83) |  | 1.27 (0.89-1.83) |  | 1.15 (0.78-1.70) |  |
|  |  |  |  |  |  |  |  |
| Genotype |  |  |  |  |  |  |  |
| GT1 |  | 1 |  | 1 |  | 1 |  |
| GT2 |  | 1.79 (0.88-3.62) |  | 1.72 (0.84-3.56) |  | 1.57 (0.70-3.53) |  |
| GT3 |  | 1.16 (0.77-1.74) |  | 1.15 (0.77-1.73) |  | 1.17 (0.76-1.79) |  |
| GT4 |  | 1.56 (1.04-2.32) |  | 1.57 (1.05-2.34) |  | 1.51 (1.00-2.27) |  |
|  |  |  |  |  |  |  |  |
| Years since HIV diagnosis |  |  |  |  |  |  |  |
| <10 |  | 1 |  | 1 |  | 1 |  |
| 10-19 |  | 0.89 (0.60-1.33) |  | 0.89 (0.59-1.33) |  | 1.11 (0.71-1.75) |  |
| 20-29 |  | 0.69 (0.42-1.12) |  | 0.68 (0.42-1.11) |  | 0.91 (0.54-1.56) |  |
| 30+ |  | 0.64 (0.32-1.29) |  | 0.64 (0.32-1.29) |  | 0.65 (0.29-1.43) |  |
|  |  |  |  |  |  |  |  |
| Years since first HCV positive test | |  |  |  |  |  |  |
| <10 |  | 1 |  | 1 |  | 1 |  |
| 10-19 |  | 1.08 (0.72-1.64) |  | 1.09 (0.72-1.65) |  | 1.14 (0.73-1.76) |  |
| 20+ |  | 1.16 (0.64-2.11) |  | 1.17 (0.65-2.12) |  | 1.16 (0.62-2.19) |  |
|  |  |  |  |  |  |  |  |
| HIV RNA viral load |  |  |  |  |  |  |  |
| <200 copies/ml |  | 1 |  | 1 |  | 1 |  |
| ≥200 copies/ml |  | 1.09 (0.56-2.11) |  | 1.12 (0.59-2.13) |  | 1.18 (0.58-2.41) |  |

### Supplementary results table 9. Unsuccessful treatment based on recent injection drug use

|  |  | With data on recent IDU, no outcome imputation^a^ |  | With data on recent IDU, with outcome imputation |  | With imputation of recent IDU variable |  | With data on recent IDU, complete case analysis |
| --- | --- | --- | --- | --- | --- | --- | --- | --- |
|  |  | aOR (95% CI) |  | aOR (95% CI) |  | aOR (95% CI) |  | aOR (95% CI) |
|  |  |  |  |  |  |  |  |  |
| Age  (10 years) |  | 1.13 (0.87-1.48) |  | 1.14 (0.88-1.49) |  | 1.04 (0.87-1.23) |  | 1.14 (0.87-1.49) |
|  |  |  |  |  |  |  |  |  |
| Sex at birth |  |  |  |  |  |  |  |  |
| Male |  | 1 |  | 1 |  | 1 |  | 1 |
| Female |  | 0.45 (0.23-0.88) |  | 0.45 (0.23-0.88) |  | 0.60 (0.39-0.93) |  | 0.52 (0.26-1.02) |
|  |  |  |  |  |  |  |  |  |
| Gay or bisexual male |  |  |  |  |  |  |  |  |
| Yes |  | 1 |  | 1 |  | 1 |  | 1 |
| No |  | 0.99 (0.59-1.66) |  | 0.98 (0.58-1.66) |  | 1.38 (0.97-1.99) |  | 1.03 (0.61-1.71) |
|  |  |  |  |  |  |  |  |  |
| Recent injection drug use |  |  |  |  |  |  |  |  |
| No |  | 1 |  | 1 |  | 1 |  | 1 |
| Yes |  | 1.67 (0.99-2.82) |  | 1.63 (0.96-2.74) |  | 1.65 (1.00-2.73) |  | 1.80 (1.06-3.07) |
|  |  |  |  |  |  |  |  |  |
| Previous HCV treatment |  |  |  |  |  |  |  |  |
| No |  | 1 |  | 1 |  | 1 |  | 1 |
| Yes |  | 1.25 (0.74-2.13) |  | 1.18 (0.69-2.03) |  | 1.24 (0.89-1.74) |  | 1.31 (0.76-2.25) |
|  |  |  |  |  |  |  |  |  |
| CD4+ cell count |  |  |  |  |  |  |  |  |
| 500+ |  | 1 |  | 1 |  | 1 |  | 1 |
| 499-350 |  | 1.13 (0.64-1.99) |  | 1.13 (0.65-1.97) |  | 1.21 (0.81-1.82) |  | 1.11 (0.61-2.01) |
| 200-349 |  | 2.04 (1.15-3.64) |  | 1.97 (1.10-3.52) |  | 2.00 (1.33-2.99) |  | 2.14 (1.19-3.88) |
| <200 |  | 1.57 (0.69-3.55) |  | 1.60 (0.71-3.60) |  | 1.85 (1.05-3.26) |  | 1.81 (0.76-4.27) |
|  |  |  |  |  |  |  |  |  |
| Possible cirrhosis |  |  |  |  |  |  |  |  |
| No |  | 1 |  | 1 |  | 1 |  | 1 |
| Yes |  | 1.03 (0.60-1.78) |  | 1.05 (0.61-1.80) |  | 1.33 (0.92-1.91) |  | 0.96 (0.54-1.70) |
|  |  |  |  |  |  |  |  |  |
| Genotype |  |  |  |  |  |  |  |  |
| GT1 |  | 1 |  | 1 |  | 1 |  | 1 |
| GT2 |  | 0.92 (0.21-3.98) |  | 0.85 (0.19-3.73) |  | 1.92 (0.93-3.99) |  | 1.03 (0.23-4.56) |
| GT3 |  | 1.11 (0.62-1.97) |  | 1.10 (0.62-1.97) |  | 1.13 (0.74-1.71) |  | 1.14 (0.64-2.03) |
| GT4 |  | 1.70 (0.90-3.22) |  | 1.65 (0.87-3.12) |  | 1.65 (1.10-2.46) |  | 1.54 (0.80-2.96) |
|  |  |  |  |  |  |  |  |  |
| Years since HIV diagnosis |  |  |  |  |  |  |  |  |
| <10 |  | 1 |  | 1 |  | 1 |  | 1 |
| 10-19 |  | 1.20 (0.65-2.21) |  | 1.22 (0.66-2.26) |  | 0.89 (0.60-1.34) |  | 1.16 (0.62-2.16) |
| 20-29 |  | 0.95 (0.47-1.90) |  | 0.92 (0.45-1.89) |  | 0.69 (0.43-1.12) |  | 0.82 (0.41-1.68) |
| 30+ |  | 1.08 (0.42-2.76) |  | 0.98 (0.36-2.65) |  | 0.66 (0.33-1.33) |  | 0.70 (0.25-1.98) |
|  |  |  |  |  |  |  |  |  |
| Years since first HCV |  |  |  |  |  |  |  |  |
| positive test |  |  |  |  |  |  |  |  |
| <10 |  | 1 |  | 1 |  | 1 |  | 1 |
| 10-19 |  | 1.07 (0.60-1.89) |  | 1.12 (0.63-1.97) |  | 1.10 (0.73-1.65) |  | 1.12 (0.64-1.97) |
| 20+ |  | 0.62 (0.24-1.65) |  | 0.68 (0.26-1.76) |  | 1.15 (0.64-2.06) |  | 0.60 (0.22-1.63) |
|  |  |  |  |  |  |  |  |  |
| HIV RNA viral load |  |  |  |  |  |  |  |  |
| <200 copies/ml |  | 1 |  | 1 |  | 1 |  | 1 |
| ≥200 copies/ml |  | 0.83 (0.32-2.17) |  | 0.84 (0.32-2.21) |  | 1.10 (0.56-2.15) |  | 0.75 (0.26-2.18) |

a. Multivariable analysis from Table 3 in manuscript

### Supplementary results table 10. SVR4+ testing complete case analysis compared to multiple imputation

|  | Multiple imputation |  | Complete case analysis |
| --- | --- | --- | --- |
|  | aOR (95% CI) |  | aOR (95% CI) |
| Age (10 years) | 0.89 (0.78-1.02) |  | 0.90 (0.78-1.05) |
|  |  |  |  |
| Population group |  |  |  |
| GBM | 1.00 |  | 1.00 |
| Male Hx of IDU | 1.28 (0.93-1.75) |  | 1.29 (0.90-1.83) |
| Female Hx of IDU | 1.19 (0.79-1.78) |  | 1.25 (0.80-1.94) |
| Male Hetero/other | 1.42 (0.99-2.04) |  | 1.48 (0.98-2.22) |
| Female Hetero/other | 1.14 (0.73-1.76) |  | 1.10 (0.66-1.82) |
|  |  |  |  |
| Previous HCV treatment |  |  |  |
| No | 1.00 |  | 1.00 |
| Yes | 0.66 (0.48-0.90) |  | 0.66 (0.47-0.92) |
|  |  |  |  |
| CD4+ cell count |  |  |  |
| 500+ | 1.00 |  | 1.00 |
| 499-350 | 0.86 (0.63-1.18) |  | 0.85 (0.60-1.21) |
| 200-349 | 1.19 (0.85-1.68) |  | 1.21 (0.84-1.75) |
| <200 | 1.34 (0.86-2.11) |  | 1.64 (1.02-2.63) |
|  |  |  |  |
| Possible cirrhosis |  |  |  |
| No | 1.00 |  | 1.00 |
| Yes | 1.13 (0.83-1.53) |  | 0.93 (0.66-1.30) |
|  |  |  |  |
| Genotype |  |  |  |
| GT1 | 1.00 |  | 1.00 |
| GT2 | 1.39 (0.73-2.63) |  | 1.41 (0.71-2.79) |
| GT3 | 1.17 (0.86-1.61) |  | 1.16 (0.83-1.62) |
| GT4 | 1.01 (0.71-1.42) |  | 1.07 (0.76-1.49) |
|  |  |  |  |
| Years since HIV diagnosis |  |  |  |
| <10 | 1.00 |  | 1.00 |
| 10-19 | 1.21 (0.89-1.64) |  | 1.27 (0.90-1.80) |
| 20-29 | 0.80 (0.55-1.19) |  | 0.89 (0.58-1.38) |
| 30+ | 1.23 (0.76-2.00) |  | 1.27 (0.73-2.18) |
|  |  |  |  |
| Years since first HCV positive test |  |  |  |
| <10 | 1.00 |  | 1.00 |
| 10-19 | 1.01 (0.73-1.38) |  | 0.99 (0.70-1.42) |
| 20+ | 1.18 (0.74-1.88) |  | 1.28 (0.77-2.13) |
|  |  |  |  |
| HIV RNA viral load |  |  |  |
| <200 copies/ml | 1.00 |  | 1.00 |
| ≥200 copies/ml | 1.98 (1.31-2.99) |  | 2.04 (1.28-3.23) |


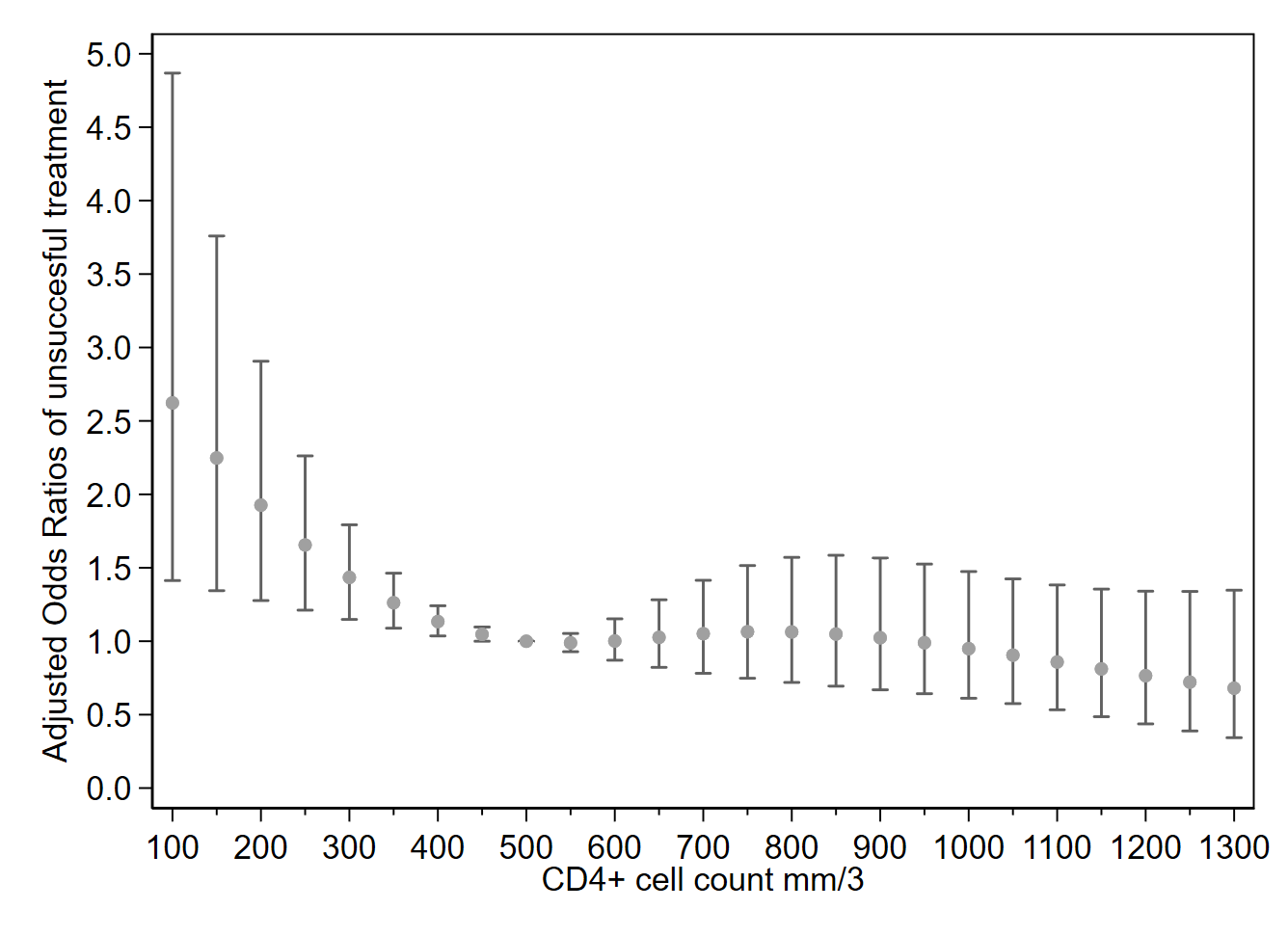


### Supplementary results figure 1. Restricted cubic spline of CD4 cell count with reference of 500 cells/mm^3^


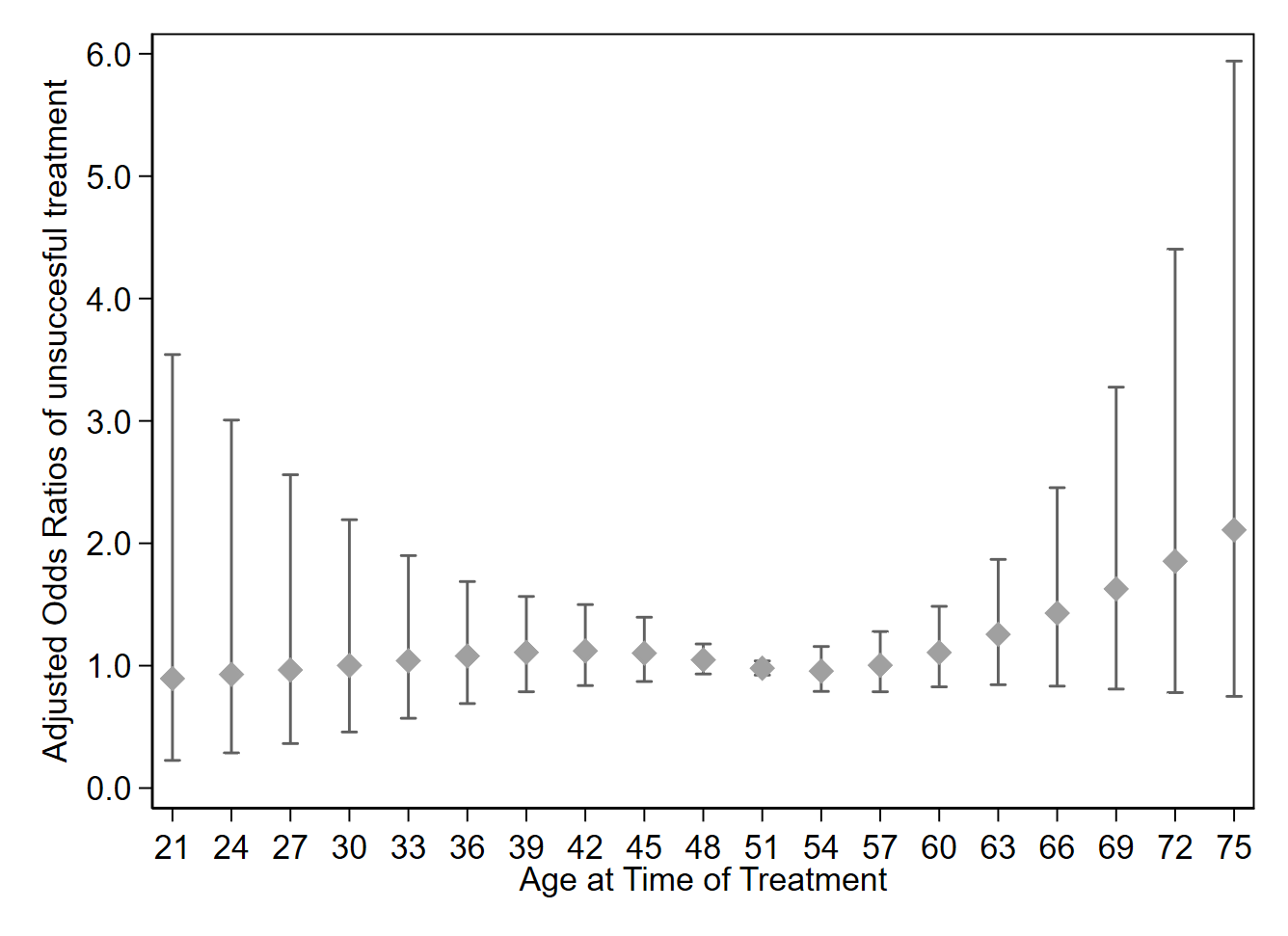


### Supplementary results figure 2. Restricted cubic spline of age with reference of 50 years


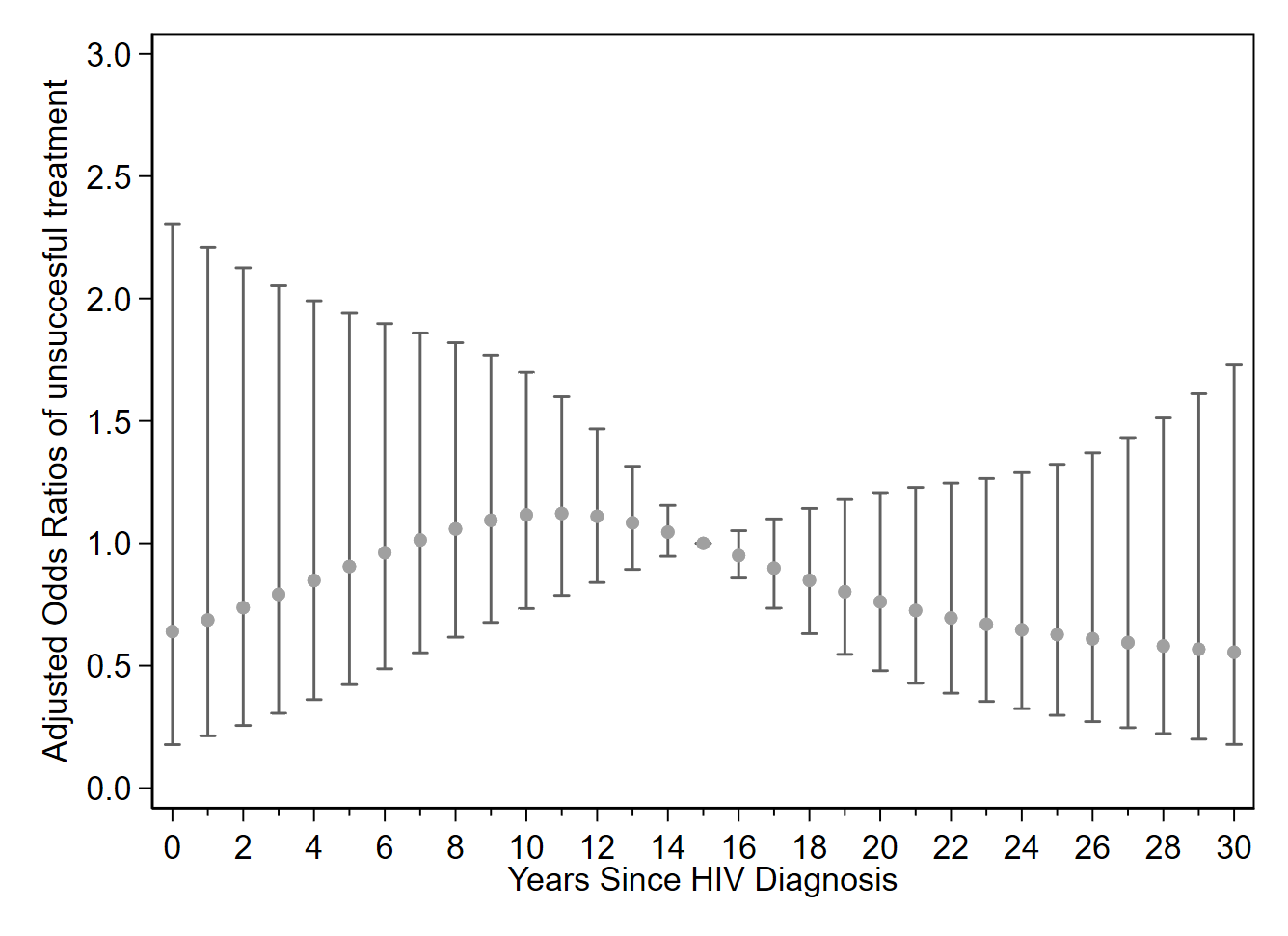


### Supplementary results figure 3. Restricted cubic spline of years since HIV diagnosis with reference of 15 years


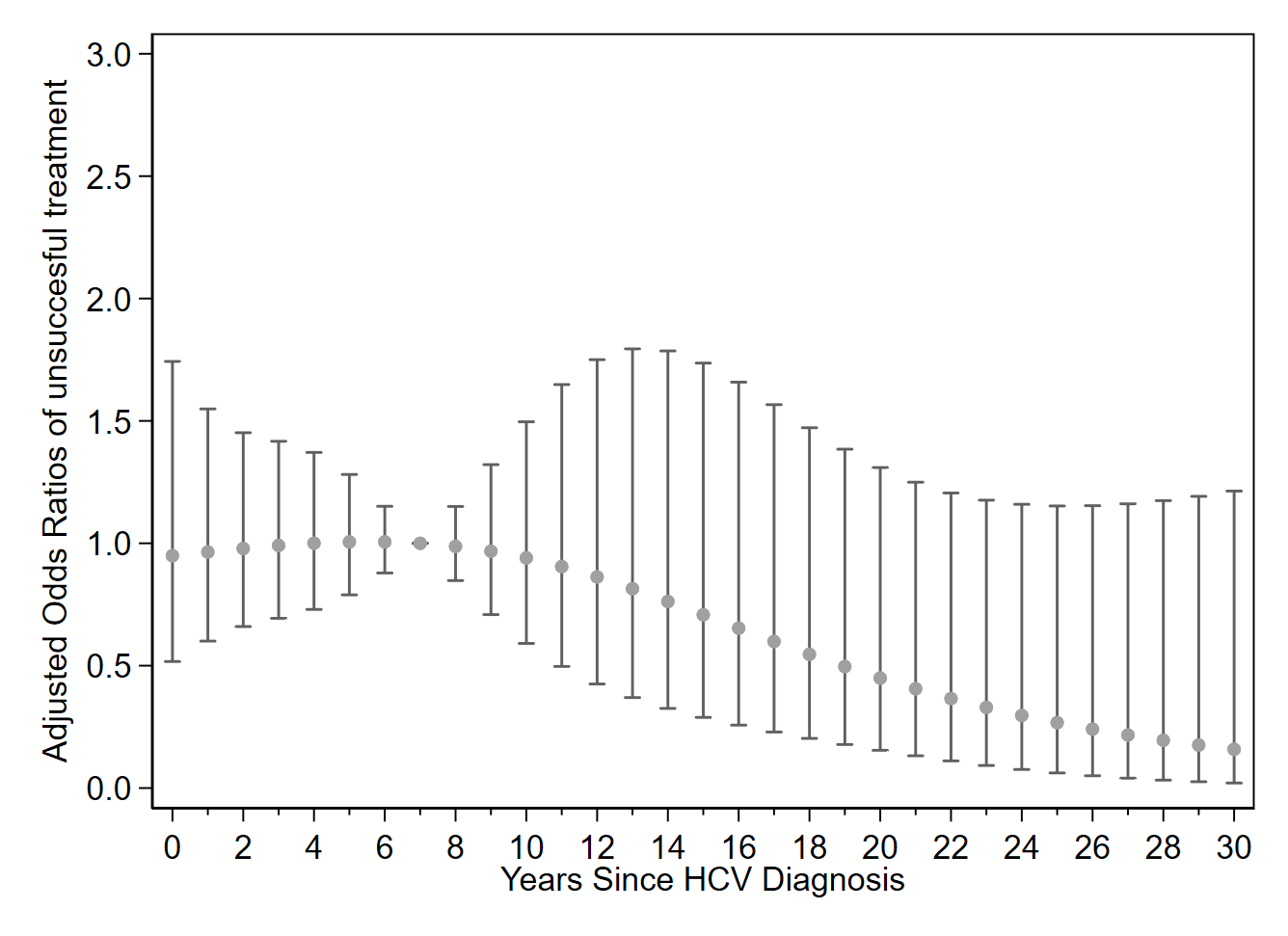


### Supplementary results figure 4. Restricted cubic spline of years since HCV diagnosis with reference of seven years

## References

1. Bui H, Zablotska-Manos I, Hammoud M, Jin F, Lea T, Bourne A, et al. Prevalence and correlates of recent injecting drug use among gay and bisexual men in Australia: Results from the FLUX study. Int J Drug Pol. 2018;55:222-30.

2. van Santen DK, Asselin J, Haber NA, Traeger MW, Callander D, Donovan B, et al. Improvements in transition times through the HIV cascade of care among gay and bisexual men with a new HIV diagnosis in New South Wales and Victoria, Australia (2012-19): a longitudinal cohort study. Lancet HIV. 2021;8(10):e623-e32.

3. European Association for the Study of the Liver Clinical Practice Guideline Panel. EASL Clinical Practice Guidelines on non-invasive tests for evaluation of liver disease severity and prognosis - 2021 update. J Hepatol. 2021;75(3):659-89.

4. Vuille-Lessard É, Rodrigues SG, Berzigotti A. Noninvasive Detection of Clinically Significant Portal Hypertension in Compensated Advanced Chronic Liver Disease. Clin Liver Dis. 2021;25(2):253-89.

## InCHEHC acknowledgments - all cohorts

| The authors thank the study participants for their contribution to the research. The authors acknowledge the contribution of the **ACCESS team** members and ACCESS advisory committee members who are not co-authors of this article. The authors also acknowledge all clinical services participating in ACCESS. The list of ACCESS team members, ACCESS advisory committee members and participating ACCESS services can be found on the ACCESS website ([https://accessproject.org.au](https://accessproject.org.au/)). ACCESS is a partnership between the Burnet Institute, Kirby Institute and National Reference Laboratory. |
| --- |
| **AQUITAINE (ANRS CO3 AQUITAINE / AquiVIH-NA)** scientific committe: P. Bellecave, P. Blanco, F. Bonnet (Chair), S. Bouchet, D. Breilh, C. Cazanave, S. Desjardin, V. Gaborieau, A. Gimbert, M. Hessamfar, L. Lacaze-Buzy, D. Lacoste, ME Lafon, E. Lazaro, O. Leleux., F. Le Marec, G. Le Moal, D. Malvy, L. Marchand, P. Mercié, D. Neau, I. Pellegrin, A. Perrier, V. Petrov-Sanchez, M.O. Vareil, L. Wittkop (Methodologist). Participating centers : Hôpital Saint André, CHU de Bordeaux, Médecine Interne et Maladies Infectieuses, (N. Bernard, F. Bonnet, D. Bronnimann H. Chaussade, D. Dondia, P. Duffau, I. Faure, M. Hessamfar, P Mercié, P. Morlat, E. Mériglier, F. Paccalin, E. Riebero, C. Rivoisy, MA Vandenhende) ; Hôpital Pellegrin, CHU de Bordeaux, Maladies Infectieuses et Tropicales, (L. Barthod, C. Cazanave, FA. Dauchy, A. Desclaux, M. Ducours, H. Dutronc, A. Duvignaud, J. Leitao, M. Lescure, D. Neau, D. Nguyen, D. Malvy, T. Pistone, M. Puges, G. Wirth) ; Hôpital Haut-Lévêque, CHU de Bordeaux, Médecine Interne et Maladies Infectieuses, (C. Courtault, F. Camou, C. Greib, E. Lazaro, JL. Pellegrin, E. Rivière, JF. Viallard) ; Hôpital d’Agen, Médecine Interne (Y. Imbert, M. Thierry-Mieg, P. Rispal) ; Hôpital de Libourne, Médecine Interne (O. Caubet, H. Ferrand, S. Tchamgoué) ; Hôpital de Bayonne, Maladies Infectieuses (S. Farbos, MO. Vareil, H. Wille); Hôpital de Dax, Médecine Interne et Maladies Infectieuses, (K. Andre, L. Caunegre, Y. Gerard, F. Osorio-Perez); Hôpital Saint-Cyr/Villeneuve-sur-Lot, Maladies Infectieuses, (I. Chossat); Hôpital de Mont de Marsan, Médecine Interne et Maladies Infectieuses, (G. Iles, Y. Gerard, M. Labasse-Depis, F. Lacassin); Hôpital d’Arcachon, Médecine Interne, (A. Barret, C. Courtault); Hôpital de Périgueux, Médecine Interne et Maladies Infectieuses, (B Castan, J. Koffi, N. Rouanes, A. Saunier, JB Zabbe); Hôpital de Pau, Médecine Interne et Maladies Infectieuses, (G. Dumondin, V. Gaborieau); Hôpital d’Orthez, Médecine Interne, (Y. Gerard) ; CHU de Poitiers, Médecine Interne et Maladies Infectieuses, (G. Beraud, G. Le Moal, M. Catroux, M. Garcia, V. Giraud, JP. Martellosio, F. Roblot) ; Hôpital de Saintes, Médecine Interne, (T. Pasdeloup) ; Hôpital d’Angoulême, Médecine Interne, (A. Riché, M. Grosset, S. Males, C. Ngo Bell) ; Hôpital de Jonzac, Maladies Infectieuses, (T. Pasdeloup), Hôpital de Saint jean d’Angely, Maladies Infectieuses, (T. Pasdeloup). Other departements : Immunology: P. Blanco, I. Pellegrin ; CRB-BBS: C. Carpentier, I. Pellegrin ; Virology: P. Bellecave, ME. Lafon, C. Tumiotto ; Pharmacology: S. Bouchet, D. Breilh, G. Miremeont-Salamé ; Data collection : D. Arma, G. Arnou, MJ Blaizeau, P. Camps, M. Decoin, S. Delveaux, F. Diarra, L. Gabrea, S. Lawson-Ayayi, E. Lenaud, D. Plainchamps, A. Pougetoux, B. Uwamaliya, K. Zara ; IT departement : V. Conte, M. Gapillout ; Project Team : O. Leleux (Project Leader), F. Le Marec (Statistitien), A. Perrier (Data Manager), website : <https://aquivih-na.fr/>. |
| The **ATHENA** database is maintained by Stichting HIV monitoring and supported by a grant from the Dutch Ministry of Health, Welfare and Sport through the Centre for Infectious Disease Control of the National Institute for Public Health and the Environment (<https://www.hiv-monitoring.nl/en/research-using-our-data/submit-research-proposal/rules-acknowledgement>). |
| **Canadian Co-Infection Cohort** (CTN222) co-investigators–Drs Lisa Barrett, Jeff Cohen, Brian Conway, Curtis Cooper, Pierre Côté, Joseph Cox, M. John Gill, Shariq Haider, Mark Hull, Valérie Martel-Laferrière, Erica E. M. Moodie, Neora Pick, Danielle Rouleau, Aida Sadr, Steve Sanche, Roger Sandre, Marie-Louise Vachon, Sharon Walmsley, and Alexander Wong. Project coordination: Isabelle Robichaud and Data management: Shouao Wang. We also acknowledge the Canadian Co-Infection Cohort participants, the study coordinators, and nurses for their assistance with study coordination, participant recruitment, and care. |
| Members of the **CEASE** study group: Protocol Steering Committee - Gail Matthews (Chair, The Kirby Institute, University of New South Wales [UNSW] Sydney, Sydney, Australia), David Baker (East Sydney Doctors, Sydney, Australia), Mark Bloch (Holdsworth House Medical Practice, Sydney, Australia), Joanne Carson (The Kirby Institute, UNSW Sydney, Sydney, Australia), Gregory Dore (The Kirby Institute, UNSW Sydney, Sydney, Australia), Joseph Doyle (Burnet Institute, Melbourne, Australia), Tim Duck (NSW Health, Sydney, Australia), Robert Finlayson (Taylor Square Private Clinic, Sydney, Australia), Margaret Hellard (Burnet Institute, Melbourne, Australia), Hayden Jose (Australasian Society for HIV, Viral Hepatitis and Sexual Health Medicine [ASHM], Sydney, Australia), Sarah Lambert (AIDS Council of NSW, Sydney, Australia), Stuart Loveday (Hepatitis NSW, Sydney, Australia), Pip Marks (The Kirby Institute, UNSW Sydney, Sydney, Australia), Marianne Martinello (The Kirby Institute, UNSW Sydney, Sydney, Australia), Jeffrey Post (The Albion Centre, Sydney, Australia), Leila Stennett (Australian Federation of AIDS Organisations, Sydney, Australia), Vanessa Towell (ASHM, Sydney, Australia), Joseph Sasadeusz (Royal Melbourne Hospital, Melbourne, Australia). Coordinating Centre, The Kirby Institute, UNSW Sydney, Sydney, Australia - Gail Matthews (Coordinating Principal Investigator), Joanne Carson (Study Coordinator), Gregory Dore (Coordinating Principal Investigator), Ecaterina Filep (Data Manager), Joanne Carson (Study Coordinator), Pip Marks (Clinical Trials Manager), Marianne Martinello (Senior Research Fellow, Statistician), Kathy Petoumenos (Statistician), Ineke Shaw (Systems Manager), Jasmine Yee (Study Coordinator), Lanni Lin (Study Coordinator). Site Principal Investigators - New South Wales: Eva Jackson (Blue Mountains Sexual Health, Sydney, Australia), Nicholas Doong (Dr Doong’s Surgery, Sydney, Australia), David Baker (East Sydney Doctors, Sydney, Australia), Mark Bloch (Holdsworth House Medical Practice, Sydney, Australia), Phillip Read (Kirketon Road Centre, Sydney, Australia), Archana Sud (Nepean Sexual Health, Sydney, Australia), Gregory Dore (St Vincent’s Hospital, Sydney, Australia), Anna McNulty (Sydney Sexual Health Centre, Sydney, Australia), Robert Finlayson (Taylor Square Private Clinic, Sydney, Australia), Jeffrey Post (The Albion Centre, Sydney, Australia), Shailendra Sawleshwarkar (Western Sydney Sexual Health, Sydney, Australia); Queensland: Diane Rowling (Brisbane Sexual Health Clinic, Brisbane, Australia); South Australia: David Shaw (Royal Adelaide Hospital, Adelaide, Australia); Victoria: Richard Moore (Northside Clinic, Melbourne, Australia). Site Coordinators - New South Wales: Vincenzo Fragomeli (Blue Mountains Sexual Health, Sydney, Australia), Shane Hewitt (Dr Doong’s Surgery, Sydney, Australia),Melissa Benson (East Sydney Doctors, Sydney, Australia), Annabelle Caspersz (Holdsworth House Medical Practice, Sydney, Australia), Rosie Gilliver (Kirketon Road Centre, Sydney, Australia), Vincenzo Fragomeli (Nepean Sexual Health, Sydney, Australia), Alison Sevehon, Fiona Peet, and Rebecca Hickey (St Vincent’s Hospital, Sydney, Australia), Ruthy McIver (Sydney Sexual Health Centre, Sydney, Australia), Ching Tan (Taylor Square Private Clinic, Sydney, Australia), Raghib Ahmad (The Albion Centre, Sydney, Australia), Holly Miller, Tichaona Jaricha (Western Sydney Sexual Health, Sydney, Australia); Queensland: Fiona Taylor (Brisbane Sexual Health Clinic, Brisbane, Australia); South Australia: Catherine Ferguson (Royal Adelaide Hospital, Adelaide, Australia); Victoria: Susan Boyd and Sian Gowalds (Northside Clinic, Melbourne, Australia. |
| The **co-EC** Study acknowledge all participants and site teams from The Alfred, Melbourne Sexual Health Centre, Prahran Market Clinic, Northside Clinic, Thorne Harbour Health and Melbourne Health; people support from the Australian National Health and Medical Research Council; and investigator initiated funding support from Bristol Myers Squibb. |
| The **CoRIS** steering committe members: Santiago Moreno, Inma Jarrín, David Dalmau, Maria Luisa Navarro, Maria Isabel Gonzalez, Federico Garcia, Eva Poveda, Jose Antonio Iribarren, Felix Gutierrez, Rafael Rubio, Francesc Vidal, Juan Berenguer, Juan Gonzalez, M Angeles Munoz-Fernandez. |
| **Scientific Committee of the ANRS CO13 HEPAVIH Study Group:** D. Salmon (co-Principal investigator), L. Wittkop (co-Principal Investigator & Methodologist), P. Sogni (co-Principal Investigator), P. Carrieri, L. Esterle (project manager), P. Trimoulet, J. Izopet, L. Serfaty, M.A. Valantin, G. Pialoux, J. Chas, K. Barange, A. Naqvi, E. Rosenthal, A. Bicart-See, O. Bouchaud, A. Gervais, C. Lascoux-Combe, C. Goujard, K. Lacombe, C. Duvivier, , D. Neau, P. Morlat, F. Bani-Sadr, F. Boufassa, , C. Solas, H. Fontaine, L. Piroth, A. Simon, D. Zucman, F. Boué, P. Miailhes, E. Billaud, H. Aumaître, D. Rey, G. Peytavin, O Zaegler, representative members of the sponsor (clinical research and pharmacovigilance department), representative members of patient organization including TRT5). |
| **MOSAIC collaborators**: J.T.M. van der Meer, R. Molenkamp, M. Mutschelknauss, H.E. Nobel, H.W. Reesink, J. Schinkel, M. van der Valk, J.W. Vanhommerig (Academic Medical Center, Amsterdam, the Netherlands); G.E.L. van den Berk, K. Brinkman, D. Kwa, N. van der Meché, A. Toonen, D. Vos (Onze Lieve Vrouwe Gasthuis, Amsterdam, the Netherlands); M. van Broekhuizen, F.N. Lauw, J.W. Mulder (MC Slotervaart, Amsterdam, the Netherlands); J.E. Arends, A. van Kessel, I. de Kroon (University Medical Center Utrecht, Utrecht, the Netherlands); A. Boonstra, M.E. van der Ende, S. Hullegie, B.J.A. Rijnders (Erasmus Medical Center, Rotterdam, the Netherlands); T.J.W. van de Laar (Sanquin Blood Supply Foundation, Amsterdam, the Netherlands); L. Gras, C. Smit (Stichting HIV Monitoring, Amsterdam, the Netherlands); A.M. Newsum, M. Prins, W. van der Veldt (Public Health Service of Amsterdam, Amsterdam, the Netherlands). |
| The authors would like to thank all clinicians and clinical research technicians participating to the **SAIDCC** database of the Infectious Diseases Unit of St Antoine Hospital, AP-HP, Paris, France: Jean-Luc Meynard, Jérôme Pacanowski, Laure Surgers, Marie-Caroline Meyohas, Dorothée Chopin, Benedicte Lefebvre, Diane Bollens, Laure Surgers, Nadia Valin, Thibault Chiarabini, Zineb Ouazene, Pauline Campa, Julie Lamarque, Rym Monard, Christian Tran. |
| Members of the **Swiss HIV Cohort Study** Abela I, Aebi-Popp K, Anagnostopoulos A, Battegay M, Bernasconi E, Braun DL, Bucher HC, Calmy A, Cavassini M, Ciuffi A, Dollenmaier G, Egger M, Elzi L, Fehr J, Fellay J, Furrer H, Fux CA, Günthard HF (President of the SHCS), Hachfeld A, Haerry D (deputy of "Positive Council"), Hasse B, Hirsch HH, Hoffmann M, Hösli I, Huber M, Kahlert CR (Chairman of the Mother & Child Substudy), Kaiser L, Keiser O, Klimkait T, Kouyos RD, Kovari H, Kusejko K (Head of Data Centre), Martinetti G, Martinez de Tejada B, Marzolini C, Metzner KJ, Müller N, Nemeth J, Nicca D, Paioni P, Pantaleo G, Perreau M, Rauch A (Chairman of the Scientific Board), Schmid P, Speck R, Stöckle M (Chairman of the Clinical and Laboratory Committee), Tarr P, Trkola A, Wandeler G, Yerly S. |
